# Environmental tuning of thyroid hormone signaling drives developmental plasticity

**DOI:** 10.1126/sciadv.aec5359

**Published:** 2026-08-07

**Authors:** Marcela Herrera, Saori Miura, Zoé Chamot, Mathieu Reynaud, Emma Gairin, Roger Huerlimann, Timothy Ravasi, Laurence Besseau, Yann Gibert, David Lecchini, Vincent Laudet

**Affiliations:** ^1^ Marine Eco-Evo-Devo Unit, Okinawa Institute of Science and Technology Graduate University, 1919-1 Tancha, Onna-son, Okinawa, 904-0495 Japan.; ^2^ PSL Research University: EPHE-UPVD-CNRS, UAR 3278 CRIOBE, BP 1013, 98729, Papetoai, Moorea, French Polynesia.; ^3^ Marine Climate Change Unit, Okinawa Institute of Science and Technology Graduate University, 1919-1 Tancha, Onna-son, Okinawa, 904-0495 Japan.; ^4^ College of Science & Engineering, James Cook University, Townsville 4814, Australia.; ^5^ Sorbonne Université, CNRS, Biologie Intégrative des Organismes Marins, BIOM, Observatoire Océanologique de Banyuls-sur-Mer, 66650 Banyuls-sur-Mer, France.; ^6^ Department of Cellular and Molecular Biology, University of Mississippi Medical Center, 2500 North State Street, Jackson, MS 39216, USA.; ^7^ Laboratoire d’Excellence CORAIL, 58 avenue Paul Alduy, 66860, Perpignan CEDEX, France.; ^8^ Marine Research Station, Institute of Cellular and Organismic Biology, Academia Sinica, YiLan, Taiwan.; ^9^ CNRS IRL 2028 “Eco-Evo-Devo of Coral Reef Fish Life Cycle” (EARLY), Okinawa Institute of Science and Technology Graduate University, 1919-1 Tancha, Onna-son, Okinawa, 904-0495 Japan.

## Abstract

Vertebrate developmental transitions unfold in environments increasingly altered by global change. Thyroid hormones, a deeply conserved endocrine axis, coordinate post-embryonic transformations, yet the effects of natural ecological variation on this signaling remain largely unexplored—despite their potential to shape developmental plasticity and acclimation to local environments. To address this, we investigated a common reef fish (*Acanthurus triostegus*), which undergoes a thyroid-dependent metamorphosis during settlement into coastal nurseries. By combining transcriptomic, hormonal, and metabolic profiling across distinct natural habitats, we show that settlement triggers a profound reprogramming of energy metabolism, tightly coupled to thyroid signaling. Strikingly, the magnitude and direction of thyroid hormone regulation varied with habitat, producing divergent metabolic and physiological states. These results demonstrate that thyroid hormones act not only as a developmental timer but as a dynamic integrator of environment and physiology. More broadly, they reveal a conserved mechanism of vertebrate developmental plasticity, highlighting endocrine flexibility as a potential buffer of population resilience under rapid environmental change.

## Teaser

Environmental cues rewire thyroid hormone signaling to drive developmental plasticity.

## INTRODUCTION

Developmental transitions are pivotal moments in vertebrate life histories, aligning internal programs with ecological and energetic demands. Central to this alignment is thyroid hormone (TH) signaling, a deeply conserved vertebrate endocrine system orchestrating post-embryonic transformations across taxa (*1*, *2*). In amphibians, they initiate tail resorption and limb development (*3*, *4*); in flatfishes, they direct eye migration and craniofacial remodeling (*5*); in salmonids, they control smoltification, facilitating adaptation for seawater entry (*6*). In reptiles, TH drive embryonic growth, influence sex differentiation through interactions with sex steroid pathways, and modulate metabolic rate (*7*). In birds, maternal TH in egg yolks first support embryonic growth and organ development, then surge at hatching to promote lung function, thermoregulation, and neuromuscular control (*8*). In mammals, TH regulate perinatal switches in metabolism, thermogenesis, and gut maturation (*9*, *10*). Together, these examples underscore the deep conservation of TH signaling as a master regulator of vertebrate development.

Although traditionally seen as an internal developmental timer, TH signaling is highly sensitive to environmental factors (*2*, *11*). Pollutants disrupt amphibian and fish metamorphosis by interfering with TH synthesis and receptor function (*12*–*14*). In teleosts, TH regulate key physiological processes, including osmoregulation and metabolic responses to fluctuations in salinity and nutrient availability (*2*, *15*). Temperature and seasonal cues further influence TH-mediated growth and the functional maturation of organ systems in mammals and birds (*16*). However, it remains unclear how natural environmental variation shapes TH function in wild populations. Elucidating these mechanisms is essential, as conserved endocrine pathways—through their flexible responses to ecological cues—may enable vertebrates to fine-tune development and physiology, enhancing adaptive capacity under accelerating global change (*17*). Evidence from humans links circulating thyroid hormone (T_3_) levels to longevity, diverse demographic traits, and socio-economic status (*18*), further reinforcing TH’s broad systemic significance across vertebrates. This raises a central question: to what extent does natural ecological variation modulate TH signaling to shape development and physiology in wild populations?

Coral reef fishes offer a powerful model to address this gap, as population persistence depends on a TH-driven metamorphosis that coincides with habitat settlement (*19*)—a developmental bottleneck closely linked to ecological context. In the convict surgeonfish (*Acanthurus triostegus*), this transition involves a remodeling of craniofacial morphology and the digestive tract, along with a trophic shift from planktonic feeding to benthic herbivory, all unfolding as larvae encounter heterogeneous coastal habitats (*19*). To investigate how environmental variation shapes TH signaling and its downstream effects, we combined transcriptomic, endocrine, and metabolic profiling across developmental stages and ecologically distinct nursery habitats in Moorea Island, French Polynesia. This integrative, systems-level approach reveals how environmental cues modulate the timing and magnitude of developmental plasticity, offering new insights into the conserved mechanisms by which TH orchestrate vertebrate life-history transitions. Specifically, we examined whether habitat diversity tunes TH signaling, how such modulation aligns with energetic demands, and its implications for environmentally sensitive developmental plasticity.

## RESULTS

### Transcriptomic insights reveal major transformations during metamorphosis

**Fig. 1.**
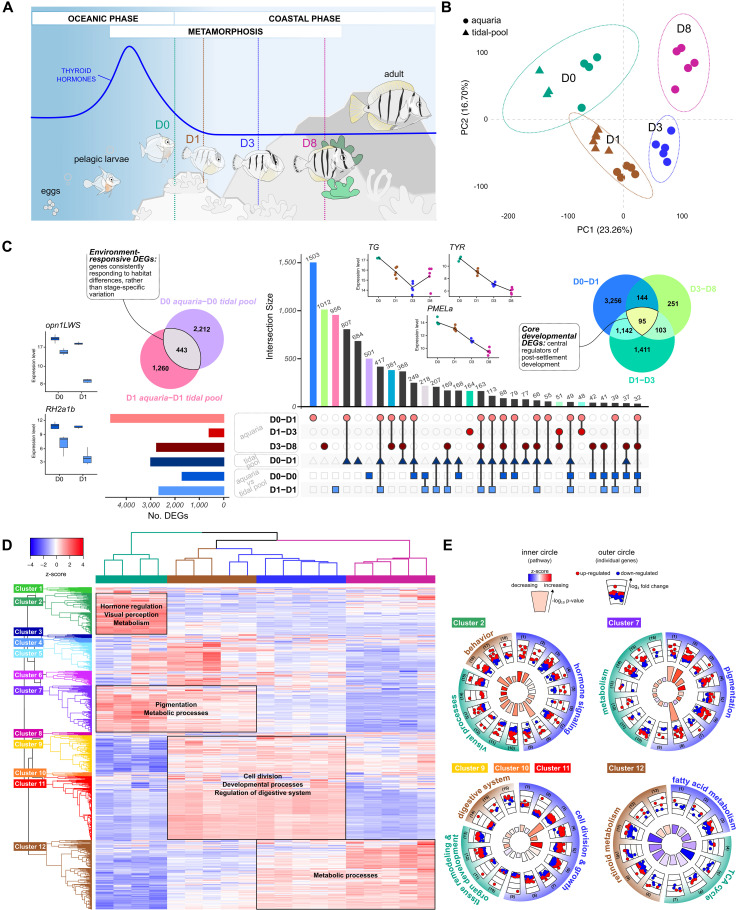
Transcriptional reprogramming during thyroid hormone-driven metamorphosis in the convict surgeonfish *Acanthurus triostegus.* (**A**) A surge in thyroid hormone signaling drives the transition from pelagic larvae (D0, green) through early post-recruitment stages at 12 hours (D1, brown), 3 (D3, blue), and 8 days (D8, pink), culminating in the juvenile phenotype. (**B**) Principal component analysis (PCA) separates samples by developmental stage (PERMANOVA *P* < 0.001; post hoc *P* < 0.01). D0 larvae were collected during a recruitment event and were reared in either aquaria (circles) or natural tidal pools (triangles). Overlap between rearing environments at D1 indicates that early transcriptomic shifts are primarily developmentally programmed rather than environmentally induced. (**C**) UpSet plot showing intersections of differentially expressed genes (DEGs) across developmental transitions in the aquarium experiment (D0 − D1, D1 − D3, D3 − D8), the D0 − D1 transition in tidal-pool larvae, and habitat-specific comparisons at D0 and D1. Vertical bars indicate exclusive intersection sizes, with connected dots showing contributing comparisons; colors correspond to the respective contrasts. Representative environment-responsive DEGs (left boxplots) show genes differing between aquaria and wild conditions at D0 (dark blue) and D1 (light blue), whereas representative developmental DEGs (right insets) show progressive expression changes across post-settlement stages. Colors match developmental stages in (A) and (B). Overlap patterns reveal a core developmental transcriptional program shared across post-settlement transitions, alongside distinct habitat-responsive genes associated with local environmental conditions during early settlement. (**D**) Clustering of the 1,000 most variable genes identifies four expression modules corresponding to successive stages of metamorphosis and major shifts in physiological state. Color intensity indicates standardized expression (z-scores): red (upregulated), blue (downregulated), white (unchanged). (**E**) Circular plots summarize enriched Gene Ontology (GO) terms for each module. Bar height indicates significance (log_10_ adjusted *P* value), inner bar color reflects mean expression, and outer scatterplots show individual DEGs. Full enrichment details are provided in table S1. Fish illustration credit: S. Vianello.

Metamorphosis in *A. triostegus* involves rapid physiological and morphological transformations as larvae recruit to reefs during lunar-synchronized pulses, orchestrated by a conserved TH signaling peak (Fig. 1A) (*19*). To capture the molecular dynamics of this process, we collected transparent larvae at the onset of reef recruitment (D0) and reared them in aquaria, sampling at 12 hours (D1), 3 days (D3), and 8 (D8) days post-settlement—key developmental stages previously described in this species (*19*). All larvae were collected during a single reef recruitment event, ensuring that individuals at D0 were of the same developmental stage. This high degree of synchrony is characteristic of *A. triostegus* metamorphosis, with settlement occurring within hours across individuals (*13*, *19*, *20*). Transcriptomic profiling revealed extensive shifts in gene expression, with the largest changes immediately after settlement (D0 − D1: 4,637 DEGs), followed by smaller transitions (D1 − D3; 593 DEGs; D3 − D8; 2,751 DEGs). Comparison with larvae reared in natural reef tidal pools confirmed that these patterns represent intrinsic developmental progression rather than rearing artifacts (Fig. 1B and fig. S1A).

Distinct gene expression signatures characterize each post-settlement stage, reflecting the physiological and metabolic reorganization accompanying metamorphosis (Fig. 1, C to E, and table S1). Within these changes, we identified core developmental DEGs, defined as genes consistently differentially expressed across all post-settlement transitions. This set included key components of TH synthesis (*TG*) and downstream TH-responsive processes such as pigmentation (*TYR*, *PMEL*), indicating sustained TH axis activity throughout metamorphosis. By contrast, environmentally responsive DEGs—genes persistently affected by habitat conditions at D0 and D1—included those enriched for circadian and visual functions (*opn1LWS*, *RH2a*). At settlement (D0), genes were enriched for hormone signaling, visual perception, and behavioral responses such as swimming and locomotion. The D0 − D1 transition involved shifts in pigmentation and energy metabolism, including nucleotide synthesis, the citric acid (TCA) cycle, insulin signaling, and triglyceride transport. Between D1 and D3, expression patterns indicated increased DNA replication and cell cycle activity, tissue remodeling, organ development, and enhanced gastrointestinal function, highlighted by a switch from early protease and lipase expression to elevated carbohydrase activity (fig. S1B). By D3 − D8, a cluster of genes associated with the TCA cycle and fatty acid metabolism exhibited altered expression, with overall activity reduced relative to earlier stages, signaling ongoing metabolic reorganization. These transcriptomic dynamics reveal a highly coordinated developmental program during metamorphosis. To uncover the regulatory drivers of these stage-specific changes, we next examined endocrine signaling—particularly TH—and their role in orchestrating the metabolic reconfiguration that underlies larval settlement and adaptation to reef habitats.

### Thyroid hormones coordinate a stage-specific metabolic reprogramming

**Fig. 2.**
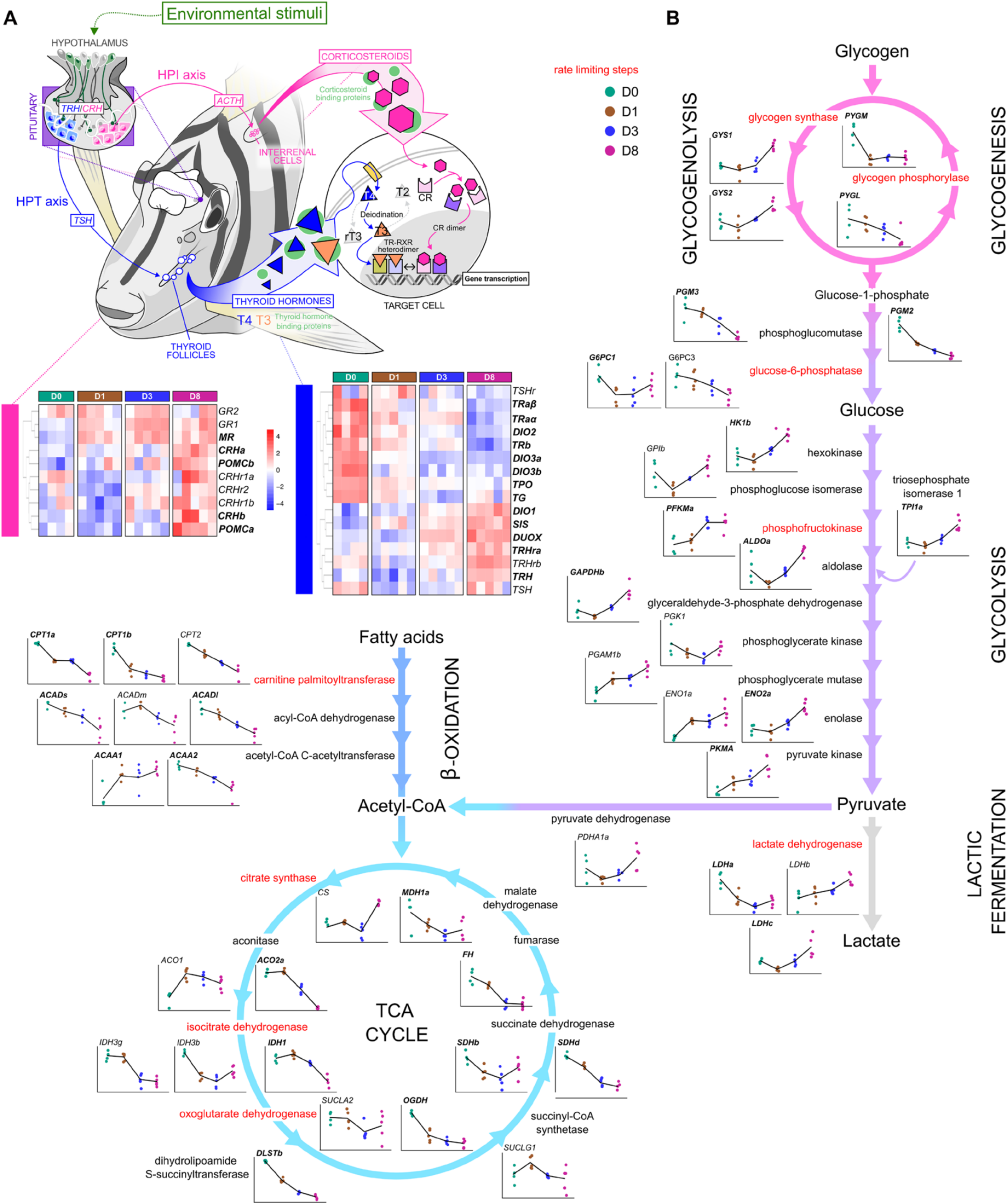
Gene expression dynamics underlying endocrine regulation and metabolic reprogramming during metamorphosis in *Acanthurus triostegus*. (**A**) Neuroendocrine integration of environmental cues in teleosts occurs through two converging axes: the hypothalamic-pituitary-thyroid (HPT, blue) and hypothalamic-pituitary-interrenal (HPI, pink) pathways. Thyrotropin-releasing hormone (*TRH*) and corticotropin-releasing hormone (*CRH*) stimulate secretion of thyroid-stimulating hormone (*TSH*) and adrenocorticotropic hormone (*ACTH*), activating thyroid follicles and interrenal cells to produce thyroid hormones (T_4_, T_3_, rT_3_) and corticosteroids. Peripheral deiodinases convert T_4_ into active T_3_ or inactive T_2_/rT_3_, enabling stage-specific transcriptional regulation via thyroid hormone receptors (TR), corticosteroid receptors (CR), and retinoid X receptors (RXR). The accompanying heatmap illustrates dynamic expression of thyroid- and corticosteroid-pathway genes across metamorphic stages. Rows represent genes; color intensity reflects standardized expression (z-scores). Temporally regulated genes are shown in bold. (**B**) Metabolic pathway dynamics parallel endocrine changes. Fish transition from a predominantly aerobic metabolic profile at the larval stage (D0) toward increased reliance on anaerobic pathways by D8. Expression trajectories are shown for genes in glycogenesis, glycogenolysis, glycolysis, lactic fermentation, fatty acid β-oxidation, and the tricarboxylic acid (TCA) cycle. Expression levels (log_2_ normalized counts +1; *y*-axis) are shown for individual fish across developmental stages (*x*-axis), with black lines indicating stage-wise means. Rate-limiting enzymes are highlighted in red and temporally regulated genes in bold. See figs. S2 to S4 for extended analysis. Fish illustration credit: S. Vianello.

Building on the transcriptomic signatures of metabolic reorganization, we profiled key genes involved in the hypothalamic-pituitary-thyroid (HPT) axis and TH signaling (Fig. 2A). Genes governing hormone synthesis and transport showed coordinated regulation: thyroglobulin (*TG*) and thyroid peroxidase (*TPO*) declined, while the sodium/iodide symporter (*SIS*) increased, reflecting shifts in iodine uptake and hormone production. Thyroid-releasing hormone (*TRH*) and thyroid-stimulating hormone (*TSH*) decreased after settlement, and deiodinases (*DIO2*, *DIO3a*, *DIO3b*) were downregulated, further modulating T_4_ activation and T_3_ catabolism. *DIO1* displayed a complex trajectory consistent with dual roles in hormone activation and degradation, while thyroid hormone receptor (TR) expression also decreased, modulating downstream responsiveness. Transcriptional coordination between HPT- and hypothalamic-pituitary-interrenal (HPI)-associated genes was also apparent: corticotropin-releasing hormone (*CRH*) expression fell after the TH peak but later increased alongside mineralocorticoid and glucocorticoid receptors (*MR*, *GR1*, *GR2*). Pro-opiomelanocortin genes (*POMCa*, *POMCb*), which encode precursors for stress, pigmentation, and appetite hormones (*21*, *22*), also showed coordinated shifts, consistent with developmental alignment between thyroid- and stress-related endocrine pathways.

Because TH are central regulators of energy metabolism (*10*), we next examined how these developmental dynamics reconfigure major metabolic pathways—glycolysis, lactic fermentation, fatty acid β-oxidation, and the TCA cycle—focusing on rate-limiting enzymes that anchor pathway control (*23*) (Fig. 2B and figs. S2 to S4). Transcriptomic profiles revealed a clear shift after settlement, marked by reduced expression of aerobic metabolism genes and heightened expression of anaerobic pathway genes. Glycolysis genes (*HK1b*, *PFKMa*) and most lactate dehydrogenases (*LDH*) increased, while fatty acid β-oxidation (*CPT1*) and TCA cycle genes (*IDH1*, *OGDH*) peaked at D0 and then declined, suggesting a developmental shift away from lipid-based energy use toward greater carbohydrate utilization. Early in settlement, when glycolytic activity was still limited, glycogenolysis genes (*PYGL*, *PYGM*) were strongly upregulated, highlighting glycogen as the primary energy source at D0. Glycogen synthesis genes (*GYS1*, *GYS2*) were comparatively less active, consistent with mobilization and depletion of glycogen reserves. At the same time, genes involved in lipid biosynthesis and fat storage decreased sharply, revealing minimal de novo lipogenesis. As development progressed, fatty acid β-oxidation became more prominent, consistent with later lipid utilization, revealing net pathway-level shifts from aerobic oxidation to anaerobic carbohydrate metabolism despite intra-pathway variation.

Together, these transcriptional patterns support a rapid metabolic reorganization at settlement, with TH dynamics closely aligned to shifting energetic demands. This endocrine-metabolic integration is consistent with the morphological and physiological transformations for the transition from pelagic larvae to reef-associated juveniles. Consistent with experimental evidence from clownfish (*24*) and *A. triostegus* (*19*), as well as groupers (*25*) and salmonids (*26*), our findings indicate that TH signaling is tightly correlated to the metabolic reprogramming that underpins teleost metamorphosis. More broadly, parallels in amphibians (*27*, *28*) and post-embryonic metabolic regulation in birds (*29*) and mammals (*10*) suggest that TH-dependent metabolic remodeling is a conserved feature of vertebrate development.

### Ecological modulation of endocrine regulation drives developmental plasticity

**Fig. 3.**
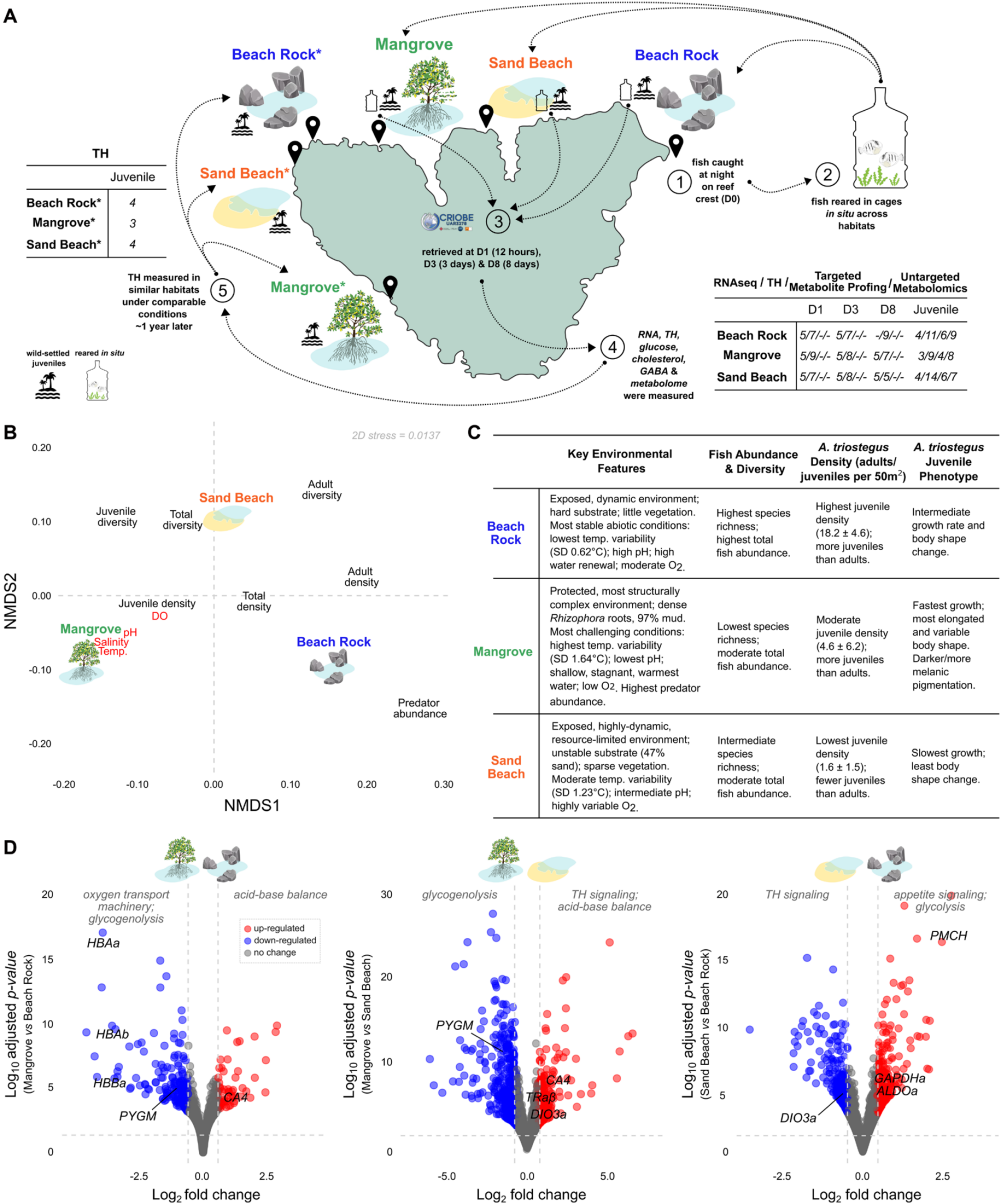
Environmental context shapes developmental plasticity in *Acanthurus triostegus*. (**A**) Experimental design combining in situ larval rearing and wild juvenile sampling across three nursery habitats: beach rock, mangrove, and sand beach. Pelagic larvae (D0) collected during a recruitment event were reared in habitat-specific cages and sampled at 12 hours (D1), 3 (D3), and 8 days (D8) post-settlement for transcriptomic and thyroid hormone (TH) analyses. Wild-settled juveniles were collected from each habitat for integrated transcriptomic, hormonal, and metabolomic analyses. A second juvenile cohort (*) collected the following year validated TH dynamics. RNA from D8 beach rock samples was excluded due to degradation. Sample sizes for each assay are shown in tables. (**B**) NMDS ordination separates habitats based on distinct abiotic (red) and biotic (black) environmental signatures (DO, dissolved oxygen; Temp., temperature). (**C**) Nursery habitats differ strongly in ecological quality: mangrove and beach rock support higher juvenile density and fish diversity, whereas sand beach represents a low-resource environment (*20*). (**D**) Volcano plots reveal habitat-specific transcriptional programs. Relative to beach rock, mangrove juveniles upregulate acid-base pathways while downregulating oxygen-transport and glycogenolytic machinery. Compared with sand beach, mangrove fish show enhanced TH modulation and ion-base balance alongside reduced glycogen mobilization. In contrast, sand beach juveniles exhibit increased appetite signaling and glycolytic metabolism relative to beach rock, coupled with reduced TH inactivation. Together, these patterns highlight environment-dependent coupling of endocrine regulation, metabolism, and physiological performance across nursery habitats.

Because TH signaling is highly sensitive to environmental variation and fuels ecological adaptation and phenotypic diversity across vertebrates (*2*, *30*), we asked how habitat modulates endocrine and metabolic regulation in *A. triostegus*. To test this, larvae were reared in situ across three ecologically distinct nursery habitats around Moorea—beach rock, mangrove, and sand beach—each imposing different constraints on growth (*20*). Individuals were sampled at D1, D3, and D8 post-settlement, alongside wild-settled juveniles for comparison (Fig. 3, A to C). Habitat exerted strong effects on gene expression across stages, producing distinct transcriptomic signatures that persisted despite the loss of D8 beach rock samples due to RNA degradation. Strikingly, juveniles from beach rock and mangrove habitats exhibited similar expression patterns despite contrasting environments, while both diverged sharply from sand beach. Differential expression analysis reflected this pattern (Fig. 3D): 236 DEGs separated beach rock and mangrove, 1,215 DEGs distinguished mangrove and sand beach, and 465 DEGs differentiated beach rock and sand beach, with beach rock fish showing an intermediate transcriptomic profile. These contrasts were driven by coordinated shifts in functional pathways, including acid-base regulation, oxygen transport, metabolic processes, and TH signaling.

**Fig. 4.**
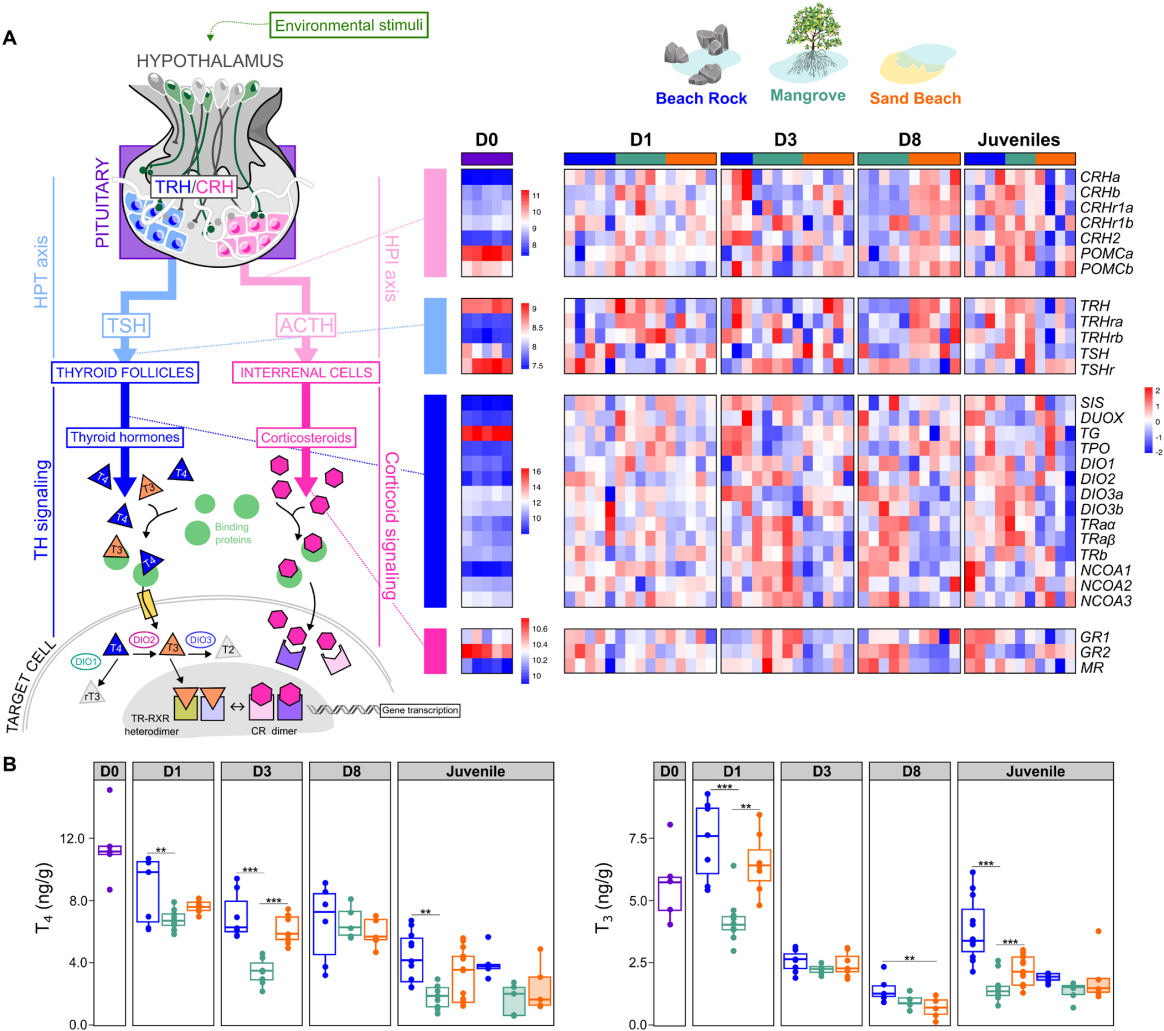
Habitat-specific endocrine tuning links environmental context to developmental trajectories in *Acanthurus triostegus*. (**A**) Environmental cues drive habitat-specific neuroendocrine regulation of thyroid and corticoid pathways. External signals regulate hypothalamic *TRH* and *CRH*, activating pituitary *TSH* and *POMC* to drive thyroid hormones (TH; T_4_, T_3_) and corticoid production. Local TH availability is further regulated by deiodinases (*DIO1–3*), converting T_4_ into active (T_3_) or inactive (T_2_, rT_3_) forms. T_3_ acts through *TR-RXR* complexes and recruitment of nuclear coactivators (*NCOA*s) to regulate transcription. Meanwhile, *ACTH* activates glucocorticoid (*GR*) and mineralocorticoid (*MR*) signaling. Heatmaps show z-score–normalized expression of key HPT and HPI genes across habitats—beach rock (blue), mangrove (green), sand beach (orange)—and developmental stages (D1, D3, D8, juveniles). D0 larvae (purple) are shown with absolute expression values. Red indicates higher expression, blue lower expression, white no change. D8 beach rock samples were excluded due to RNA degradation. (**B**) Whole-body T_4_ and T_3_ concentrations (ng/g) across developmental stages. D0 larvae are shown in purple; shaded box plots represent a second wild cohort sampled one year later. TH levels follow habitat-specific trajectories, with highest concentrations in beach rock, intermediate levels in sand beach, and lowest in mangrove. Asterisks indicate significant differences among habitats (**P < 0.01, ***P < 0.001; Tukey HSD post hoc following one-way ANOVA). These results show that local ecological context tunes endocrine dynamics, linking TH signaling to habitat-dependent developmental and metabolic plasticity.

TH regulation showed strong habitat-specific variation, detectable as early as D1 and intensifying by D8 (Fig. 4A). We note that D1 and D3 showed greater variability within habitats; our analyses therefore focused on D8 and juveniles, when habitat-specific trajectories stabilized. In mangrove fish, HPT axis activity declined sharply, with reduced *TRH*, *TSH*, and TH synthesis/transport genes (*SIS*, *DUOX*, *TPO*, *DIO2*) but increased *DIO3*, TR, and *NCOAs*, consistent with modified regulation of TH pathways at the whole-organism level. This persisted in juveniles, which had the lowest circulating TH (T_4_: 1.80 ± 0.69 ng/g; T_3_: 1.49 ± 0.59 ng/g) and reduced HPI activity. Beach rock juveniles, in contrast, showed low central HPT signaling but elevated expression of downstream components (*SIS*, *DUOX*, *DIO2*, TR), consistent with the highest TH levels (T_4_: 5.71 ± 2.70 ng/g; T_3_: 5.02 ± 2.34 ng/g). Sand beach fish were intermediate (T_4_: 3.49 ± 1.85 ng/g; T_3_: 2.18 ± 0.57 ng/g) with generally lower TH-related gene expression. Importantly, repeated measurements a year later confirmed these habitat-specific TH signatures (Fig. 4B). While second-year comparisons were not statistically significant, the patterns mirrored the first-year cohort, revealing a decoupling of TH levels from HPT axis activity and flexible, context-dependent endocrine regulation across habitats.

### Habitat-specific metabolic reprogramming mirrors endocrine variation

**Fig. 5.**
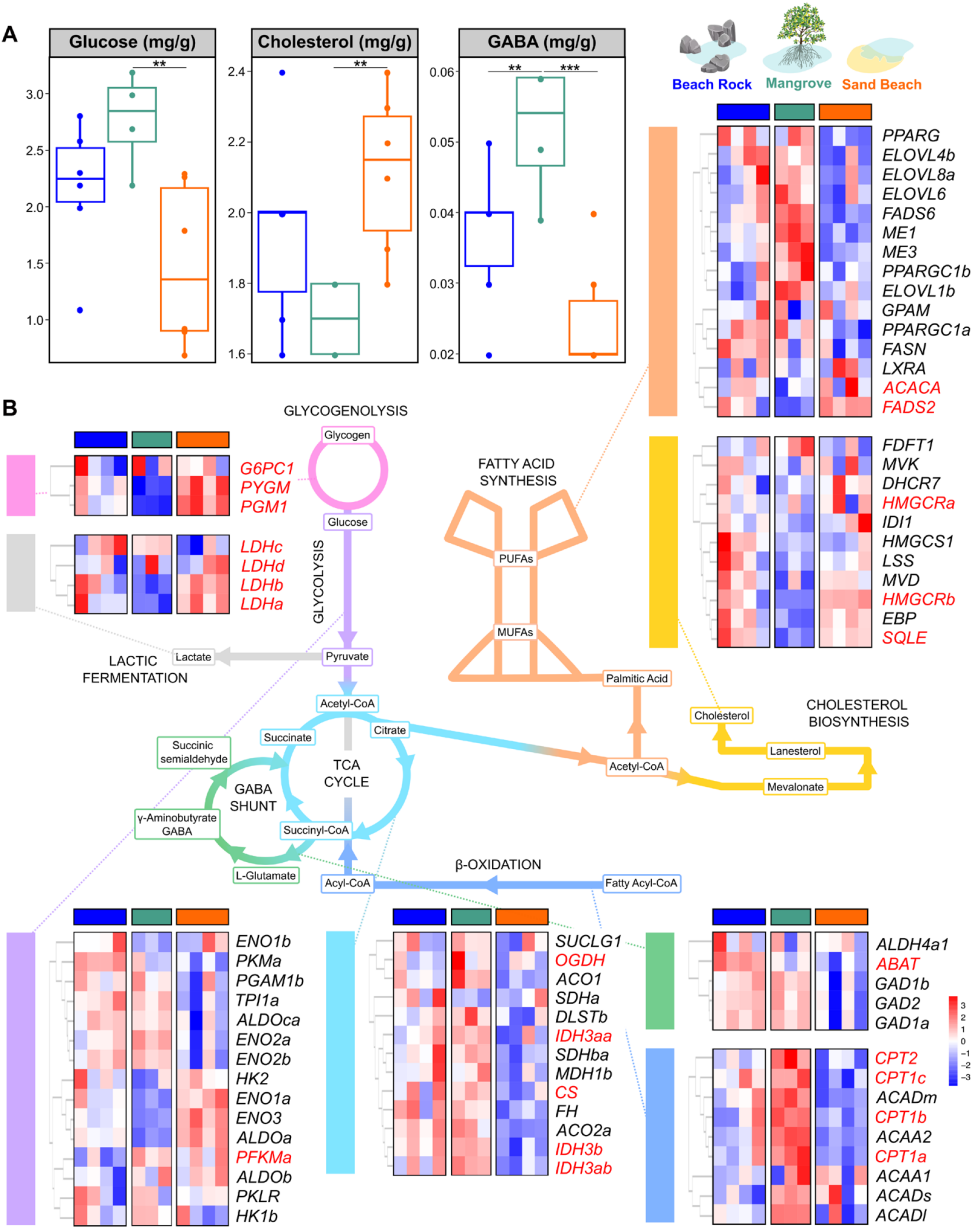
Habitat-specific metabolic remodeling in wild-settled *Acanthurus triostegus* juveniles. (**A**) Whole-body concentrations of glucose, cholesterol, and GABA (mg/g) in juveniles from three ecologically distinct nursery habitats: beach rock (blue), mangrove (green), and sand beach (orange). Asterisks indicate significant differences among habitats (**P < 0.05, ***P < 0.01; Tukey HSD post hoc following one-way ANOVA). (**B**) Schematic overview of major metabolic pathways—including glycolysis, glycogenolysis, lactic fermentation, the TCA cycle, fatty acid β-oxidation, fatty acid synthesis, cholesterol biosynthesis, and the GABA shunt. Heatmaps show z-score–normalized expression of key genes across habitats, with red indicating high expression and blue low, and white no change. Rate-limiting enzymes are highlighted in red, marking critical regulatory control points within each pathway.

To test whether hormone variation translates into physiological differences, we profiled metabolic gene expression and key metabolites—glucose, cholesterol, and γ-aminobutyric acid (GABA)—in wild-settled juveniles across the three habitats (Fig. 5A), and further characterized broader metabolic shifts using untargeted GC-MS metabolomics. Glucose reflects short-term energy metabolism, cholesterol indicates longer-term changes in lipid metabolism and steroidogenic capacity (*31*), and GABA—a central neurotransmitter and TCA cycle-linked metabolic intermediate—links metabolic state to TH-dependent neuroendocrine signaling (*32*, *33*). Because in situ-reared fish were isolated from natural factors such as predation, competition, and habitat complexity, which can influence metabolism independently of TH signaling, our analyses focused on juveniles developed entirely in the wild, capturing ecologically relevant patterns.

Metabolic profiles closely mirrored endocrine variation across habitats (Fig. 5B; see also fig. S5). Mangrove juveniles upregulated glycolysis, TCA cycle, and fatty acid β-oxidation genes, consistent with elevated tissue glucose (2.78 ± 0.43 mg/g) and GABA (0.05 ± 0.01 mg/g). Notably, increased expression of GABA shunt genes (*ABAT, ALDH5A1, GAD1*, *GAD2*) suggests an additional route for glucose-derived carbon into the TCA cycle, supporting aerobic energy production (*33*). Fatty acid synthesis genes (*ME*, *ELOVL*s, *FADS6*, *PPARGC1*) were also upregulated, reinforcing aerobic metabolism and lipid remodeling, while cholesterol biosynthesis genes (*DHCR7*, *HMGCR*, *MVD*, *SQLE*) were downregulated, matching the lowest cholesterol levels across habitats (1.70 ± 0.12 mg/g). By contrast, sand beach juveniles exhibited strong upregulation of glycogenolysis, glycolysis, and lactic fermentation genes, yet had the lowest glucose (1.49 ± 0.73 mg/g) and GABA (0.03 ± 0.01 mg/g), reflecting anaerobic utilization of internal energy stores, alongside the highest cholesterol levels (2.12 ± 0.23 mg/g) consistent with upregulated cholesterol biosynthesis genes. Beach rock juveniles displayed intermediate metabolic profiles, with gene expression and metabolite levels—glucose (2.17 ± 0.60 mg/g), GABA (0.04 ± 0.01 mg/g), and cholesterol (1.95 ± 0.28 mg/g)—falling between mangrove and sand beach values (Fig. 5A), consistent with their intermediate endocrine phenotype. Although absolute differences remained within a 2-fold range, shifts of comparable magnitude have been shown to alter metabolism and TH signaling in fish (*34*, *35*), supporting the biological relevance of these habitat-specific metabolic and neuroendocrine responses.

**Fig. 6.**
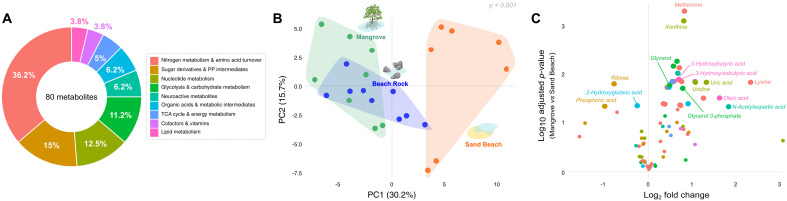
System-level metabolomics reveals habitat-specific metabolic organization. (**A**) Untargeted GC-MS metabolomics profiling of an independent cohort identified 80 water-soluble metabolites distributed across nine functionally defined categories, encompassing nitrogen metabolism & amino acid turnover, glycolysis & carbohydrate metabolism, pentose phosphate (PP) intermediates & sugar derivatives, nucleotide metabolism, TCA cycle & energy metabolism, organic acids & metabolic intermediates, lipid metabolism, neuroactive metabolites, and cofactors & vitamins. (**B**) Principal component analysis (PCA) separates metabolomes by habitat (PERMANOVA *P* < 0.001). Mangrove (green) and sand beach (orange) juveniles occupy opposite ends of metabolic space, with beach rock (blue) individuals intermediate. Each point represents an individual; shaded regions highlight group clustering. This separation reflects distinct metabolic organization associated with habitat-specific physiological strategies. (**C**) Volcano plot showing differential metabolite abundance between sand beach and mangrove juveniles. Each point represents a metabolite and is colored according to the same functional categories shown in (A). Point size reflects statistical significance, with larger points indicating metabolites meeting the significance threshold (FDR < 0.05) and smaller points indicating non-significant metabolites. Selected metabolites with large fold changes or high statistical significance are labeled. Patterns of differential abundance span multiple functional categories, consistent with distinct metabolic strategies across habitats.

Untargeted GC-MS metabolomics on an independent cohort of wild-settled juveniles from the same habitats, profiling 80 water-soluble metabolites across 9 functional categories (Fig. 6A), further supported this habitat-specific metabolic gradient. Metabolomic profiles clearly distinguished the three habitats, with mangrove and sand beach juveniles occupying opposite ends of the metabolic space and beach rock juveniles intermediate (Fig. 6B). Differentially abundant metabolites spanned multiple categories, from energy substrates and amino acids to neuroactive compounds and lipid intermediates, underscoring the breadth of habitat-driven metabolic divergence. Thirty metabolites differed significantly between sand beach and mangrove juveniles (Fig. 6C), with the majority elevated in sand beach, including glycolytic intermediates (fructose 1-phosphate, glycerol, glycerol 3-phosphate), amino acids (methionine, lysine, arginine, cysteine, ornithine), lipid metabolites (oleic acid, 3-hydroxybutyric acid, 3-hydroxyisobutyric acid), nucleotide catabolism products (xanthine, uric acid, uridine), and the cofactor pantothenic acid. This pattern is consistent with elevated substrate mobilization and metabolic turnover in sand beach juveniles, suggesting high energy expenditure potentially linked to a protein-rich diet and active foraging in a physically demanding environment. In contrast, mangrove juveniles—inhabiting a structurally complex, nutrient-rich environment with reduced predator pressure—display a metabolic signature consistent with more stable energy availability and sustained, lower-intensity metabolism.

Collectively, these results demonstrate that natural habitat differences shape TH signaling during early development and program juvenile metabolism. By linking environmental variation to endocrine regulation and metabolic outcomes, our findings reveal a mechanism through which ecological context drives developmental plasticity, enabling fish—and potentially other vertebrates—to fine-tune physiology and life-history trajectories in the wild (*1*, *2*, *30*).

### Multidimensional hormonal plasticity enables local acclimation

*TRH* and *CRH*, beyond their canonical roles in HPT and HPI activation, coordinate broader physiological responses. *TRH-*mediated TH signaling governs basal metabolism, development, and thermal adaptation by regulating temperature-responsive genes, including heat shock proteins and key metabolic enzymes (*2*, *36*). TH and corticosteroids further enhance hypoxia tolerance by regulating metabolic rate and oxygen consumption (*36*), and support osmoregulation via Na^+^/K^+^-ATPases, chloride channels, and aquaporins (*2*, *37*, *38*). *CRH* additionally stimulates *POMC* expression, increasing α*-MSH* production and modulating appetite and energy balance through melanocortin signaling (*21*, *22*). To capture this integrative endocrine control, we expanded our analysis to include genes involved in oxidative stress, hypoxia adaptation, osmoregulation, and appetite control (fig. S6), representing key axes of adaptation across habitats varying in temperature, oxygen, salinity, and food availability (Fig. 3, B and C).

Endocrine-mediated osmoregulatory and appetite responses diverged sharply among habitats (fig. S6). Mangrove and beach rock juveniles showed similarly high expression of key osmoregulatory genes (Na^+^/K^+^-ATPases, aquaporins, *IGF-1*, *GH* and its receptor, and corticosteroid receptors), despite larger salinity fluctuations in sand beach and beach rock habitats. Sand beach juveniles, in contrast, showed a weaker response. Similarities between mangrove and beach rock fish likely reflect the combined effect of multiple stressors and elevated *TRH* and *CRH* activity, potentially supporting flexible osmoregulatory capacity beyond salinity regulation. Upregulation of acid-base transporters in sand beach and beach rock fish matched the higher and more variable pH, suggesting coordinated ion-pH regulation under fluctuating conditions. Appetite-regulatory profiles further revealed contrasting metabolic strategies. Mangrove juveniles simultaneously upregulated orexigenic (appetite-stimulating; *AGRP*, *NPY*, *PMCH*) and anorexigenic (appetite-suppressing; *MC4R*, *CCK*, *CART*) genes, reflecting flexible dual regulation that may enable rapid behavioral and metabolic adjustments in a variable environment. Sand beach juveniles, on the other hand, showed elevated *LEP* and anorexigenic genes (*NMU*, *PYY*), consistent with enhanced satiety and an energy-conserving strategy that could be suited to stable but resource-limited conditions.

Endocrine-regulated responses to hypoxia and oxidative stress also diverged across environments (fig. S6). Mangrove juveniles showed the strongest upregulation of hypoxia signaling genes—including *HIF1A*, prolyl hydroxylases (*EGLN1–3*), and downstream effectors such as *VEGFA*, *ARNT*, and apoptosis regulators (*BCL2*, *BNIP3*)—consistent with the low and variable oxygen levels characteristic of this habitat. Surprisingly, antioxidant defenses were most elevated in sand beach fish, despite mangrove experiencing the highest temperature and greatest thermal variability. Key reactive oxygen species (ROS)-detoxifying genes (*PRDX*, *GPX*) and the master redox regulator *NFE2L2* were upregulated, indicating strong oxidative stress responses driven by habitat-specific pressures beyond temperature. These results suggest that local environmental variation shapes endocrine and metabolic plasticity, integrating TH and corticosteroid signaling to modulate metabolism and stress responses—illustrating how ecological context can influence vertebrate endocrine networks and potentially impact physiological performance across habitats.

## DISCUSSION

Our study reveals the molecular and physiological choreography by which TH orchestrate metamorphosis in the coral reef fish *A. triostegus*, aligning internal developmental programs with the ecological challenges of settlement. Using a multi-omics approach, we show that TH dynamics are closely associated with rapid changes in pigmentation, vision, dentition, and gut remodeling within days of reef entry (*19*), suggesting an important coordinating role during metamorphosis. Future studies involving experimental manipulation of TH signaling will be important to directly test its regulatory role in these developmental processes. TH-driven metabolic reprogramming redirects energy to meet juvenile functional demands, highlighting a central role in facilitating ecological success during this critical life-stage transition (*2*). Our results demonstrate that TH-mediated developmental plasticity allows juvenile fish to tune physiology to the diverse and fluctuating conditions of nursery habitats, complementing earlier morphological and behavioral observations (*19*).

Metamorphosis involves a profound metabolic shift from pelagic to benthic life. Larvae rely on energy-rich fatty acid β-oxidation to sustain prolonged swimming, whereas juveniles increasingly depend on glycolysis to fuel rapid tissue remodeling and adaptation to their new habitats. This shift aligns with the Gill-Oxygen Limitation Theory (GOLT) (*39*), which posits that gill surface area cannot keep pace with oxygen demands as body size increases. Within this framework, larvae in well-oxygenated open waters maintain high aerobic metabolism via the TCA cycle and fatty acid β-oxidation. By contrast, juveniles in structurally complex, oxygen-limited coastal habitats rely more strongly on glycolysis and anaerobic pathways to rapidly meet energy demands, while remaining dependent on mitochondrial metabolism for sustained ATP production (*40*). Importantly, the concordance between aquarium-reared and natural-settled larvae highlights the ecological relevance of our findings and emphasizes that developmental outcomes are dynamically shaped by local environmental conditions.

**Fig. 7.**
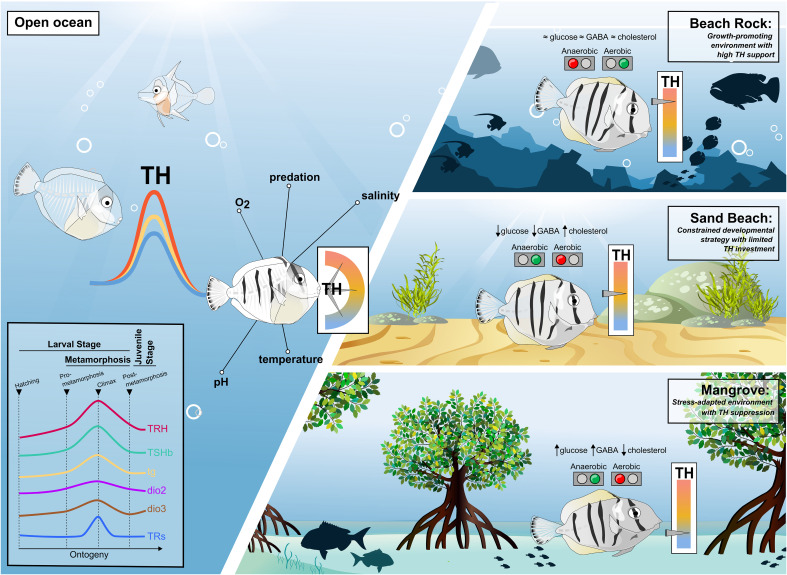
Thyroid hormones integrate ecology and development to program reef fish phenotypes. Thyroid hormones (TH) act as integrators of environmental cues and internal developmental programs, orchestrating physiology, morphology, and behavior during post-settlement metamorphosis in the convict surgeonfish *Acanthurus triostegus*. Juveniles from distinct habitats exhibit divergent TH-driven strategies: beach rock—highest TH, metabolic and transcriptomic profiles supporting rapid growth; sand beach—intermediate TH, low plasticity, anaerobic metabolism, low glucose and GABA, high cholesterol, slow growth; mangrove—lowest TH, elevated aerobic metabolism, high glucose and GABA, reduced cholesterol, darker pigmentation, elongated body shape. TH thus aligns the endocrine state with ecological context to produce habitat-specific developmental outcomes. Fish illustration credit: S. Vianello.

Comparisons across mangrove, beach rock, and sand beach nurseries reveal how environmental variation drives phenotypic diversity, influencing growth, morphology, and endocrine profiles. These patterns arise not from fixed genetic differences but the dynamic interplay between local ecological factors—temperature, oxygen availability, predation risk, and resource levels—and TH signaling, enabling juveniles at the same developmental stage to follow distinct, habitat-specific trajectories (Fig. 7). Mangrove habitats, despite their physiological challenges, support rapid growth and high morphological variability; sand beaches impose a conservative, slow growth strategy; beach rock nurseries occupy an intermediate niche (*20*). Our longitudinal approach captured developmental progression, emphasizing the value of temporal resolution over static snapshots and the importance of ecologically grounded research to reveal how natural habitats shape endocrine regulation and phenotypic diversity. More broadly, these findings highlight a conserved vertebrate principle: local environments tune hormonal pathways to generate flexible, context-dependent phenotypes, illustrating how ecological cues shape developmental plasticity across life histories (*2*, *30*).

Underlying this variability is a highly flexible endocrine system capable of fine-scale allostatic regulation (*41*). Subtle environmental differences influenced not only hormone secretion but also transport and tissue sensitivity, producing divergent endocrine profiles across habitats. Echoing earlier work showing that temperature (*13*, *42*), pollutants (*14*, *19*), and salinity (*2*, *43*) can alter TH signaling in aquatic species, the gradients we observed—lowest in mangrove, intermediate in sand beach, and highest in beach rock—reflect ecological pressures rather than systemic regulation alone. Importantly, circulating TH levels showed consistent patterns across repeated measurements one year apart and across multiple sites with comparable environmental conditions, highlighting the robustness of these endocrine signatures. Peripheral control mechanisms, including shifts in activating and deactivating enzymes, enable context-specific modulation of TH action. Mangrove fish, for example, maintained upregulation of central HPT regulators despite low TH levels, suggesting energy-saving strategies reminiscent of compensatory responses observed in other ectotherms (*42*). In contrast, sand beach juveniles showed little regulatory plasticity, consistent with a conservative, low-risk strategy, whereas beach rock juveniles exhibited high TH with tight feedback regulation supporting rapid growth. The decoupling of gene expression from hormone levels between mangrove and beach rock fish highlights the multiple regulatory layers that confer endocrine plasticity, pointing to peripheral control as an important mechanism by which the thyroid axis adjusts to ecological pressures and tunes developmental outcomes in variable coastal habitats (*2*, *17*, *30*).

This endocrine flexibility extends into metabolism and appetite control. By integrating metabolomic and transcriptomic data, we show that TH-mediated developmental plasticity extends into core processes of energy balance and feeding (*2*, *17*, *30*, *36*, *42*), interacting with neurotransmitter systems such as GABA (*32*) to couple endocrine state with appetite and stress resilience. Mangrove juveniles follow a growth-focused metabolic strategy: high glucose, low cholesterol, elevated insulin gene expression, along with strong appetite stimulation, suggest rapid carbohydrate turnover but may increase susceptibility to impaired satiety signaling (*21*). At the same time, elevated GABA and activation of hypoxia pathways enhance thermal and oxygen tolerance (*44*–*46*), promoting resilience and energy efficiency under the challenging, variable mangrove environment. Sand beach juveniles adopt a conservative, storage-oriented strategy, with low glucose, high cholesterol, reduced aerobic capacity, low GABA, and suppressed appetite—optimizing survival in resource-limited conditions. Untargeted metabolomics further supports this picture, revealing broad elevation of amino acids, lipid intermediates, and nucleotide catabolism products in sand beach juveniles, consistent with increased protein turnover and lipid mobilization under energetic constraint. Beach rock juveniles display an intermediate phenotype, balancing moderate levels of glucose, cholesterol, and GABA with upregulated cholesterol synthesis and GABA-related genes, reflecting fine-tuned TH-GABA regulation suited to the comparatively stable and favorable beach rock habitat—a pattern similarly reflected in their intermediate metabolomic profile.

Several factors temper interpretation of our findings, including individual feeding history influencing appetite-related gene expression. Nonetheless, habitat-specific TH signatures were robust, consistent across repeated measurements one year apart and across multiple sites with comparable conditions, highlighting the reliability of these endocrine patterns. Juvenile fish do not maintain a fixed physiological state but instead adjust energy allocation and endocrine activity to match the demands of their local environment. By situating TH-mediated developmental plasticity in natural contexts, our study provides insights into how endocrine networks translate environmental cues into adaptive phenotypes, offering a model for similar processes in other reef fishes and, potentially, other vertebrates. Such flexibility is critical during early ontogeny, when environmental signals interact with hormone and neurotransmitter pathways to shape long-term phenotypic trajectories (Fig. 7). While plasticity can enhance ecological performance under favorable conditions, mismatched endocrine responses may produce suboptimal phenotypes under stress. Overall, these findings illustrate how TH-mediated endocrine plasticity integrates development, metabolism, and environmental responsiveness to support adaptive life-history transitions, exemplifying a conserved vertebrate principle: local ecological conditions tune endocrine networks to generate flexible, context-dependent phenotypes that shape developmental trajectories and adaptive potential across vertebrate life histories.

## MATERIALS AND METHODS

### Ethics statement

This study did not involve endangered or protected species and was conducted in accordance with the French Polynesia code for animal ethics and scientific research. All procedures were approved by the CRIOBE-IRCP animal ethics committee (approval no. DL-20150214), and every effort was made to minimize animal suffering.

### Model species

The convict surgeonfish *A. triostegus* is a widespread coral reef species occurring across the Indo-Pacific and Eastern Pacific, including Moorea, French Polynesia (*19*, *20*, *47*, *48*). This species is a well-studied model for teleost metamorphosis (*2*, *13*, *19*). Following a ∼53-day pelagic larval phase, larvae settle on reefs and undergo rapid metamorphosis over ∼1 week, involving dramatic morphological, physiological, and behavioral changes regulated by thyroid hormones (TH) (*19*).

### Study period and sites

This study was conducted between October 2022 and March 2023 on Moorea Island, French Polynesia (S17°32′16.4589″, W149°49′48.3018″). Moorea is surrounded by a 61 km barrier reef and a 0.8–1.3 km-wide lagoon. Its fringing reefs encompass diverse environmental conditions shaped by sediment composition, freshwater input, and the presence of mangrove trees, algae, and corals (*20*). These coastal ecosystems have been classified into distinct habitat types that serve as critical nurseries for reef fishes, including *A. triostegus* (*20*). For this study, we focused on three nursery habitat types: beach rock, mangrove, and sand beach. Beach rock habitats are dominated by eroded coral slabs (> 60% bare substrate), mangroves by mud (97%) and *Rhizophora stylosa* roots, and sand beaches by sand and macroalgae with minimal vegetation. Environmental conditions vary among habitats: mangroves experience the highest and most variable temperatures (SD = 1.64°C), as well as the lowest mean pH and dissolved oxygen, while beach rock (SD = 0.62°C) and sand beach (SD = 1.23°C) are more moderate. Salinity is similar across habitats but more variable in beach rock and sand beach. Fish communities also differ, beach rock supports the highest species richness and abundance (116.7 ± 4.9 individuals per 50 m^2^), whereas mangroves have the lowest. Predator abundance is generally low, highest in mangrove (0.7 per 50 m^2^), followed by beach rock (0.6 per 50 m^2^) and sand beach (0.1 per 50 m^2^). Further details are provided in (*20*).

### Environmental and community characterization of nursery habitats

Differences among nursery habitats were visualized using non-metric multidimensional scaling (NMDS) based on a combined dataset of biotic and abiotic variables measured at each site. Biotic data comprised fish community composition (species abundances) obtained from standardized underwater visual censuses previously described in (*20*), conducted at the same habitats and locations sampled in this study. Abiotic variables included temperature, salinity, pH, and dissolved oxygen. All variables were standardized to account for differences in scale. NMDS was performed using Bray-Curtis dissimilarities in two dimensions (k = 2) with 20 random starts to ensure convergence. The analysis was carried out using the “metaMDS” function in the “vegan” package (*49*), and the best solution was selected based on the lowest stress value (stress = 0.0137; Fig. 3B).

### Fish collection and experimental design

Settlement-stage *A. triostegus* (fully transparent larvae, defined as D0) were collected at night using hand nets as they entered the reef crest on the northeast coast of the island (S17°29′49.7362″, W149°45′13.899″) during the week of the new moon. Larvae were divided into two experimental groups: in the first, fish were transferred to aquaria at the CRIOBE Marine Research Station; in the second, fish were placed in in situ cages within the three nursery habitats (beach rock, mangrove, and sand beach; Fig. 4). Tidal-pool comparisons for D0 and D1 were performed to validate aquaria transcriptomic profiles against natural conditions. Larvae were monitored continuously to protect against nocturnal predation, which precluded extending this comparison to later time points (D1 − D3). Fish were sampled at 12 hours (D1), 3 days (D3), and 8 days (D8) post-capture, wild juveniles naturally settled in each habitat collected at dusk for comparison. For the aquarium experiment, sample sizes were 4 for D0, 5 for D1, 5 for D3, and 5 for D8. For the tidal-pool comparisons, sample sizes were 3 for D0 and 6 for D1. Time points reflect days post-capture (dpc), not absolute age at reef entry (*20*). Fish were fed coral rubble with algal turf. For sampling, fish were euthanized with an overdose of MS-222 (200 mg/liter, immersion) and preserved in RNAlater (ThermoFisher Scientific) at −20°C for RNA sequencing, or dry-frozen for TH quantification and metabolite profiling. In the aquarium experiment, RNA sequencing was performed at each time point. In the in situ experiment, fish were analyzed for RNA sequencing, TH levels, and metabolites (glucose, cholesterol, GABA). Sample sizes are detailed in Fig. 4. To verify reproducibility of TH measurements, additional juveniles were collected a year later (October 2024) from comparable habitats at different sites. Additional samples for untargeted metabolomics were collected in January 2026 from the same three nursery habitats to capture broader metabolic profiles under comparable environmental conditions. All juvenile fish used for RNA-seq, TH measurements, targeted metabolite profiling, and untargeted metabolomics were measured for standard length prior to sampling, and approximate age estimates were determined for each fish based on habitat-specific growth trajectories (*20*) (fig. S7), allowing us to account for size- and age-related variation across analyses.

### RNA extraction and sequencing

Total RNA was extracted from whole larvae using the Maxwell RSC simplyRNA Tissue Kit (Promega, USA) on the Maxwell RSC instrument, following the manufacturer’s instructions. RNA quantity and quality were assessed with an Invitrogen QuBit Flex Fluorometer and Agilent TapeStation 4200. Libraries were prepared using the NEBNext Ultra II Directional RNA Library Prep Kit (New England Biolabs, USA) and sequenced on an Illumina NovaSeq6000 platform (151 bp paired-end reads) at the Okinawa Institute of Science and Technology (OIST) Sequencing Center.

### Genome and hi-C sequencing

A single adult *A. triostegus* was collected at the OIST Marine Science Station (February 26, 2021), euthanized with MS-222 (200 mg/liter), and dissected. Heart and liver tissue were snap-frozen in liquid nitrogen for genome and Hi-C sequencing. High-molecular weight DNA was extracted using the Nanobind Tissue Big DNA Kit (Circulomics, USA) with a TissueRuptor II (Qiagen, Germany). Approximately 15 μg of DNA was size-selected (30–80 kb) using BluePippin (Sage Science, USA) for Oxford Nanopore Technologies (ONT) long-read sequencing. Libraries were prepared with the Ligation Sequencing Kit 1D (SQK-LSK109, ONT), including DNA repair, end-repair/A-tailing, and adapter ligation (NEBNext modules, New England Biolabs, USA), then sequenced on an ONT MinION using three R9.4.1 flow cells across four 48-hour runs.

Hi-C libraries were generated from liver tissue following Phase Genomics Proximo Hi-C 2.0 Kit protocols (WA, USA). Tissue was minced, cross-linked with 1% formaldehyde for 20 min, quenched with 125 mM glycine for 15 min, flash-frozen, ground, and stored at −80°C. Libraries were sequenced on an Illumina NovaSeq 6000 (151 bp paired-end reads). For transcriptome sequencing, tissues (brain, eye, gill, muscle, stomach, spleen, and portions of heart and liver) were preserved in RNAlater and RNA extracted with the Maxwell RSC simplyRNA Tissue Kit. Only samples with RIN > 7.0 were used. Libraries were prepared from 400 ng total RNA using the NEBNext Ultra II Directional RNA Library Prep Kit and sequenced (151 bp paired-end) on an Illumina NovaSeq6000 at OIST.

### Chromosomal-level genome assembly

ONT reads were base-called using the super-accurate (SUP) model in Guppy v5.0.11 (*50*). Adapters were trimmed with Porechop v0.2.4 (https://github.com/rrwick/Porechop) using default parameters. High-quality reads were assembled with Flye v2.9 (*50*), specifying a genome size of 650 Mb and performing three assembly iterations. The resulting draft assembly was polished to improve accuracy using Racon v1.4.3 (https://github.com/isovic/racon) and Medaka v1.4.3 (https://github.com/nanoporetech/medaka) with the “r941_min_high_g303” model. ONT reads were then mapped back to the polished assembly using minimap2 v2.17 (*51*) and read-depth histograms were generated with Purge Haplotigs v1.0.4 (*52*); no haplotigs required purging. Genome characteristics were evaluated using a k-mer frequency analysis performed with KAT v2.4.2 (*53*) (k = 21) on quality-filtered ONT reads. The k-mer spectrum showed a dominant homozygous peak at ∼14× coverage and a secondary heterozygous peak at approximately half coverage, consistent with a diploid genome. Based on the homozygous peak, the estimated genome size was 640.3 Mb and the heterozygosity rate was estimated at 0.48%. The k-mer-based genome size estimate informed assembly parameter selection (fig. S8). Final scaffolding to achieve chromosome-level resolution was performed by Phase Genomics (WA, USA). Assembly quality and completeness were assessed using Quast v5.0.2 (*54*) and BUSCO v4.1.2 (*55*) with the actinopterygii_odb10 database (fig. S9). Base-level consensus accuracy and k-mer completeness were further evaluated using Merqury v1.3 (*56*) (k = 21) based on Illumina-derived k-mers. Analyses were performed separately for the chromosome-scale assembly (all 24 chromosomes combined) and for unassembled contigs. The chromosomal assembly showed a consensus quality value (QV) of 33.0 with an estimated error rate of 4.96 × 10^−4^ and a k-mer completeness of 91.5%, whereas unassembled contigs exhibited substantially lower completeness (1.25%) and reduced consensus quality (QV = 29.0), indicating that the vast majority of genomic sequence content is captured within chromosome-scale scaffolds (table S2).

### Prediction of gene models and functional annotation

Repetitive elements were identified using a combination of de novo and homology-based approaches. RepeatModeler v2.0.1 (*57*) with the “LTRStruct” option was first used to identify repeat families, followed by RepeatMasker v4.1.1 (*58*), which screened for known elements using both the RepeatModeler output and the Dfam v3.3 vertebrata library (*59*). Resulting annotation files were validated, merged, and filtered for redundancy using GenomeTools v1.6.1 (*60*). Gene prediction was performed with BRAKER v2.1.6 (*61*), integrating transcriptomic and protein evidence. Chromosome sequences were soft-masked prior to gene annotation using the maskfasta tool from BEDTools v2.30.0 (*62*) with the “soft” option. Transcriptomic reads from eight tissues (brain, eye, gill, muscle, stomach, spleen, heart, and liver) were trimmed with Trimmomatic v0.39 (*63*) using the parameters “TruSeq3-PE.fa:2:30:10:8:keepBothReads LEADING:3 TRAILING:3 MINLEN:36”, and then aligned to the genome with HISAT v2.2.1 (*64*) using the “dta” option. SAM files were converted to BAM format with SAMtools v1.10 (*65*).

For protein evidence, we used 571,282 manually curated sequences from UniProtKB/Swiss-Prot (*66*) (downloaded April 1, 2024) along with the proteomes from 10 teleost species retrieved from NCBI: spiny chromis damselfish (*Acanthochromis polyacanthus*: 36,657), false clownfish (*Amphiprion ocellaris*: 48,690), copperband butterflyfish (*Chelmon rostratus*: 33,962), zebrafish (*Danio rerio*: 88,942), large yellow croaker (*Larimichthys crocea*: 95,483), Nile tilapia (*Oreochromis niloticus*: 64,100), medaka (*Oryzias latipes*: 47,715), rainbow fish (*Poecilia reticulata*: 45,720), tiger puffer (*Takifugu rubripes*: 49,550), and Atlantic salmon (*Salmo salar*: 110,142). Only gene models supported by transcriptomic or protein evidence or showing homology to Swiss-Prot proteins (*66*) or Pfam domains (*67*) (identified using Diamond v2.0.9 (*68*) and InterProScan v5.48.83.0 (*69*), respectively) were retained. Functional annotation was performed using BLAST v2.10.0+ (*70*) against the NCBI non-redundant (*nr*) database with parameters “-evalue 1e-5 -max_target_seqs 1 -outfmt 6”. Gene Ontology (GO) terms were assigned using BLAST results in combination with the NCBI “gene2go.gz” and “gene2accession.gz” files (https://ftp.ncbi.nlm.nih.gov/gene/DATA/). The resulting annotation comprised 26,310 predicted-coding genes, of which 24,264 (92.2%) showed significant similarity to proteins in the *nr* database, 20,477 (77.8%) contained at least one Pfam domain, and 20,024 (76.1%) were associated with GO terms. The completeness of the final gene annotation was assessed using BUSCO v4.1.4 (*55*) with the “actinopterygii_odb10” lineage dataset.

### Transcriptomic analysis

For all samples, RNA was extracted from whole-body larvae across all groups. RNAseq libraries yielded 104–127 million reads per sample for aquarium-reared fish and 59–84 million reads for cage-reared fish and wild-settled juveniles from three habitats. Raw sequence quality was assessed using FastQC v.0.11.3 (https://www.bioinformatics.babraham.ac.uk/projects/fastqc/), and reads were trimmed using Trimmomatic v0.39 (*63*) with the parameters described above. Trimmed reads were aligned to the chromosome-scale genome using STAR v2.7.9a (*71*) with default settings. To account for RNA degradation observed in some samples, aligned reads were normalized using DegNorm v0.1.4 (*72*), correcting for transcript degradation and sequencing depth. Key sequencing and alignment metrics for each sample are summarized in table S3. Variance-stabilized counts (VST) from DESeq2 v1.26.0 (*73*) were log_2_-transformed for unsupervised analysis, including principal component analysis (PCA) using the R function “prcomp”. Differences in gene expression across developmental stages (D0, D1, D3, D8) and habitats (beach rock, mangrove, sand beach) were evaluated using permutational multivariate analysis of variance (PERMANOVA) via the “adonis2” function in the “vegan” R package (*49*). Analyses were based on a Euclidean distance matrix of VST expression values, with developmental stage and habitat specified as explanatory factors, and significance was assessed using 10,000 permutations. The top 1,000 most variable genes across time points were visualized in a heatmap, with genes clustered by Pearson correlation and developmental stages clustered by Euclidean distance. Differential expression analysis was performed using DESeq2, employing negative binomial generalized linear models with Wald significance tests, adjusting for library size and gene dispersion. Genes with adjusted *P* values <0.05 and absolute log_2_ fold change ≥0.58 were considered differentially expressed. To visualize overlaps among DEGs across key biological comparisons, including developmental transitions in aquarium-reared larvae (D0 − D1, D1 − D3, D3 − D8), the D0 − D1 transition in tidal-pool larvae, and habitat-specific differences at D0 and D1, we generated an UpSet plot using the “UpSetR” R package (*74*). This approach highlights both core developmental DEGs consistently changing across post-settlement transitions and environmentally responsive DEGs influenced by habitat conditions. Gene Ontology (GO) enrichment among differentially expressed genes was assessed using topGO (*75*), considering terms with *P* < 0.05 and occurring at least five times. Results were visualized using the “GOplot” R package (*76*), using circular plots to display z-scores (inner ring) and gene expression scatterplots (outer ring) for enriched GO terms. Building on the curated gene framework from Herrera *et al.* (*77*) and our prior expertise, we identified genes involved in thyroid hormone signaling, corticoid synthesis, and key metabolic pathways for targeted analyses in this study.

### qPCR validation of habitat-specific metabolic gene expression

To provide orthogonal support for the RNA-seq results, the expression of five metabolic genes (*ACAA2*, *ACADm*, *FADS2, LDHa*, and *ME1*) was measured by RT-qPCR in wild-settled juveniles from beach rock, mangrove, and sand beach habitats. These individuals are the same as those used for transcriptomic profiling. Specific primers for each target gene were designed based on the *A. triostegus* genomic sequence (table S4). RPL7 and POLD2 were used as reference genes, as previously validated in this species (*19*). Extracted RNA from each juvenile was converted to cDNA using the PrimerScript RT-PCR Kit (Takara Bio, Shiga, Japan). Primer efficiency and specificity were confirmed prior to expression analyses (table S4). RT-qPCR was performed using PrimeScript transcriptase (Takara Bio) with SYBR Green chemistry. Primer sequences and gene-specific cycling conditions are provided in table S4. Relative expression (RE) was calculated using the Pfaffl method (*78*)

$$\text{RE}=\frac{E{\left(\text{target}\right)}^{\Delta \text{Ct}(\text{target})}}{\surd \left[{E(\text{RPL}7)}^{\Delta \text{Ct}(\text{RPL}7)}\times {E(\text{POLD}2)}^{\Delta \text{Ct}(\text{POLD}2)}\right]}$$

where *E*(*x*) is the amplification efficiency of gene x, and ΔCt(*x*) is the difference between the sand beach group mean Ct and the individual sample Ct for gene *x*. The geometric mean of the two reference genes was used for normalization. Statistical differences in relative expression across habitats were assessed by one-way ANOVA followed by Tukey HSD post-hoc test, with a harmonic mean correction for unequal group sizes.

### Thyroid hormone quantification

Thyroid hormones (TH) were extracted from dry-frozen whole fish following the protocol of (*23*). Briefly, samples were homogenized in distilled water with a ball mill (ShakeMaster NEO, Hirata, Japan) and steel beads. Homogenates were transferred to polypropylene tubes and spiked with isotope-labeled internal standards (^13^C6-T_4_, ^13^C6-T_3_, and ^13^C6-rT_3_). Proteins were denatured with acetonitrile, followed by a 30 min equilibration at room temperature in the dark. Samples were centrifuged at 3,000 rpm for 3 min, and the supernatant was decanted into new tubes and diluted with distilled water. Extracts were loaded onto Oasis MCX cartridges preconditioned with methanol, distilled water, and 1% acetic acid. After washing with water and methanol, TH were eluted with a methanol:water:ammonia solution (70:30:1, v/v/v), evaporated to dryness, and reconstituted in methanol:water:pyridine (40:60:1, v/v/v). Hormone concentrations (T_4_, T_3_, rT_3_) were quantified by LC-MS/MS using selected reaction monitoring (SRM): m/z 777.8/731.6 for T_4_, 651.9/605.8 for T_3_, and 651.9/507.7 for rT_3_. Statistical differences among environments within each developmental stage were evaluated using one-way ANOVA followed by Bonferroni-corrected pairwise *t* tests.

### Targeted metabolite profiling

For glucose and γ-aminobutyric acid (GABA) analysis, ∼20 mg of frozen whole-body homogenate was extracted. Succinic acid-d4 was added as an internal standard, followed by 160 μl ultrapure water and 40 μl methanol. Samples were homogenized with a TissueLyser (Qiagen, Germany) for 20 s at 20 Hz, then supplemented with 160 μl of chloroform and shaken for 5 min. After addition of 400 μl of ultrapure water and another 5-min shaking step, samples were centrifuged at 16,000*g* for 5 min, and the aqueous phase was collected. Extracts were further purified by adding 300 μl ultrapure water, vortexing for 30 s, and centrifuged again. A 100 μl aliquot of the aqueous layer was frozen and lyophilized (Tomy CC-105 centrifuge; Labconco FZ-Compact vacuum lyophilizer). The dried residue was resuspended in 120 μl of 20 mg/ml 2-methoxyamine hydrochloride in pyridine and incubated at 37°C for 90 min (1,200 rpm, Eppendorf thermomixer, Germany). For derivatization, 100 μl MSTFA was added, followed by 30 min shaking at 1,200 rpm and 37°C. After centrifugation (16,000*g* for 5 min), supernatants were analyzed by gas chromatography-tandem mass spectrometry (Shimadzu GCMS-TQ8030). Procedural blanks were processed alongside samples. Peak detection and quantification were performed with GCMS Solution software (Shimadzu, Kyoto, Japan). Compounds were considered detected if (i) the analyte/internal standard peak area ratio exceeded 1,000 or (ii) a peak co-eluted with the reference standard. For analytes with multiple peaks due to derivatization, peak areas were summed. Quantification was based on a one-point calibration. Signals below twice the blank were considered not detected.

For cholesterol, ∼10 mg of frozen tissue was homogenized with zirconia beads, cholesterol-d7 (internal standard), and 100 μl ethanol using a TissueLyser (20 Hz, 20 s). Following homogenization, 100 μl of 0.5 mol/liter potassium hydroxide in ethanol was added, and samples were saponified at 50°C for 60 min in a thermomixer. Lipids were partitioned with 250 μl of hexane and 500 μl ultrapure water, shaken for 30 s, and centrifuged at 16,000*g* for 3 min. The organic (hexane) phase was collected, and a second extraction (200 μl hexane) was performed on the aqueous phase. Combined organic extracts were adjusted to 500 μl with hexane. A 100 μl aliquot was dried by centrifugal evaporation and resuspended in 200 μl tetrahydrofuran for GC-MS/MS analysis. Operational blanks were included. Peak detection, identification, and quantification followed the same criteria as above. Cholesterol was considered detected if the peak area exceeded 1,000 and co-eluted with the reference standard. Quantification was performed by calculating peak area ratios relative to the internal standard using one-point calibration. Signals below twice the blank value were considered not detected.

### Untargeted metabolomics

For untargeted metabolite profiling, ∼20 mg of freeze-ground tissue was transferred to a tube containing zirconia beads. 2-isopropylmalic acid was added as an internal standard, followed by 160 μl ultrapure water and 400 μl methanol. Samples were homogenized with a TissueLyser (Qiage, Germany) for 20 s at 20 Hz, then supplemented with 160 μl chloroform and shaken for 5 min. After addition of 400 μl ultrapure water and another 5-min shaking step, samples were centrifuged at 16,000*g* for 5 min, and the aqueous phase was collected. Extracts were further purified by adding 300 μl ultrapure water, vortexing for 30 s, and centrifuging again at 16,000*g* for 5 min. A 100 μl aliquot of the aqueous layer was recovered, concentrated by centrifugal evaporation for 40 min, and lyophilized. A pooled QC sample was prepared at this stage by combining equal volumes of the aqueous fraction from each sample and analyzed 12 times throughout the run to assess analytical stability. The dried residue was resuspended in 100 μl of 20 mg/ml methoxyamine hydrochloride in pyridine and incubated at 37°C for 90 min at 1,200 rpm. Subsequently, 50 μl MSTFA was added and silylation was performed at 37°C for 30 min at 1,200 rpm. After centrifugation at 16,000*g* for 5 min, supernatants were analyzed by gas chromatography-tandem mass spectrometry. A procedural blank was processed alongside samples throughout.

Detected metabolites were manually assigned to nine functional categories based on known biochemical roles: glycolysis & carbohydrate metabolism (sugar phosphates and glycolytic intermediates); TCA cycle & energy metabolism (organic acids directly fueling mitochondrial respiration); lipid metabolism (fatty acids, hydroxy-fatty acids, and lipid intermediates); amino acid turnover & nitrogen metabolism (amino acids and their derivatives); nucleotide metabolism (purine and pyrimidine catabolism products); neuroactive metabolites (compounds with known roles in neurotransmission or neuromodulation); organic acids & metabolic intermediates (carboxylic acids linking major metabolic pathways); sugar derivatives & pentose phosphate (PP) intermediates (products of the pentose phosphate pathway and sugar modifications); and cofactors & vitamins (essential enzymatic cofactors and water-soluble vitamins). To assess habitat-specific metabolic variation, PCA was performed on metabolite abundances using “prcomp” in R. Overall differences in metabolite abundance across the three habitats were assessed using one-way ANOVA with Benjamini-Hochberg FDR correction. Pairwise differential abundance between habitats were tested using Welch *t* tests, also with Benjamini-Hochberg correction, and metabolites with FDR < 0.05 were considered significant.
