# Supplementary material for: Environmental tuning of thyroid hormone signaling drives developmental plasticity
Source: Sci Adv. 2026 Aug 7;12(32):eaec5359. doi: 10.1126/sciadv.aec5359 (PMC13450213; doi:10.1126/sciadv.aec5359)
Supplement: Supplementary file 1 [file sciadv.aec5359_sm.pdf]

Science Advances
AAAS

Supplementary Materials for

Environmental tuning of thyroid hormone signaling drives developmental plasticity

Marcela Herrera *et al.*

Corresponding author: Marcela Herrera, marcela.herrerasarrias@gmail.com;
Vincent Laudet, vincent.laudet@oist.jp

Sci. Adv. **12**, eaec5359 (2026)
DOI: 10.1126/sciadv.aec5359

The PDF file includes:

Figs. S1 to S9
Legends for tables S1 to S4

Other Supplementary Material for this manuscript includes the following:

Tables S1 to S4

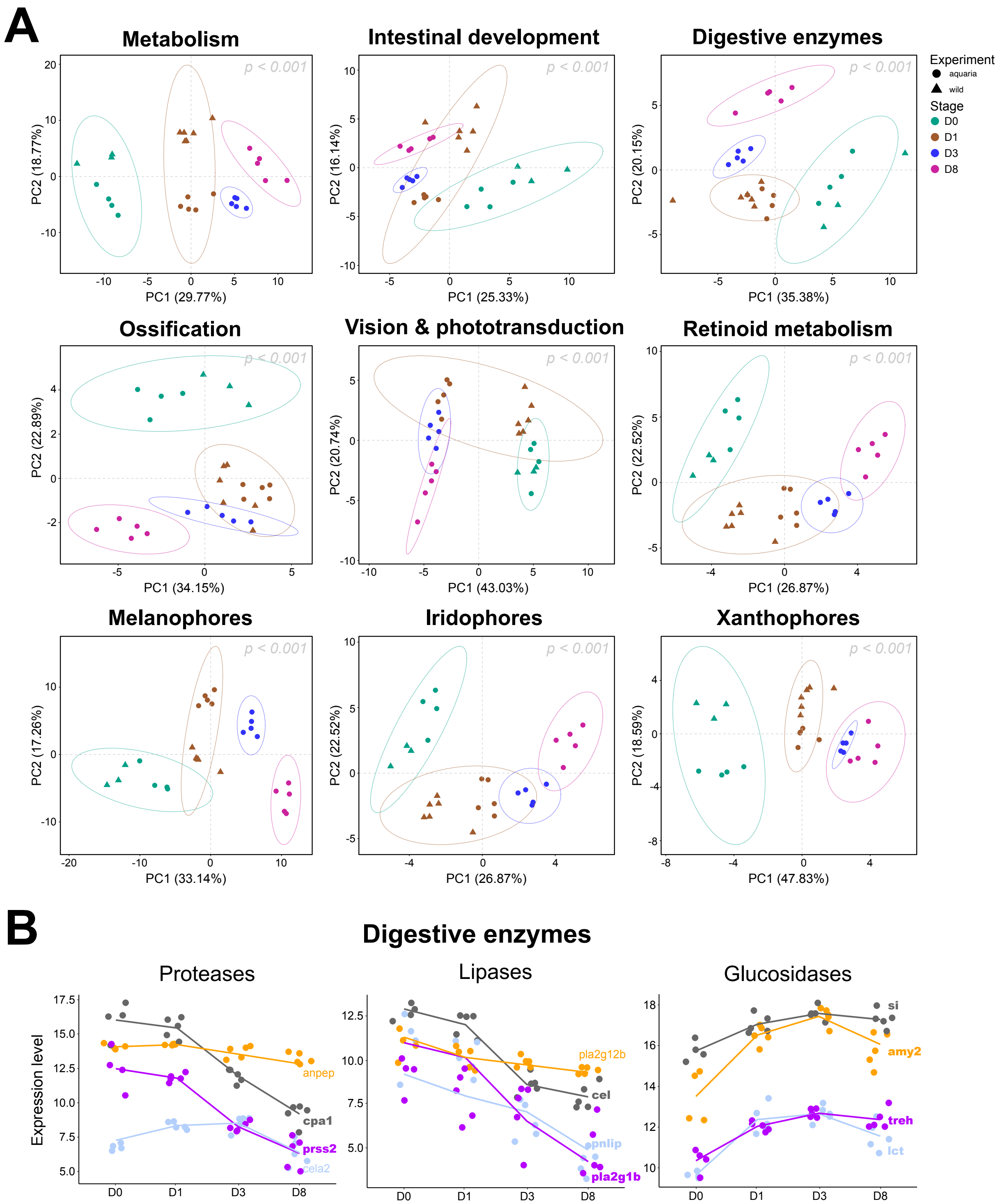

Fig. S1. Transcriptomic dynamics reveal morphological and physiological changes during post-settlement development in *Acanthurus triostegus*. (**A**) Principal component analysis (PCA) of variance-stabilized transcriptomes highlights stage-specific shifts in metabolism, intestinal development, digestive enzymes, ossification, vision/retinoid pathways, and pigment cell types (melanophores, iridophores, xanthophores) across four developmental stages (green – D0, brown – D1, blue – D3, pink – D8). Circles indicate fish reared in aquaria; triangles represent fish reared

in natural reef tidal pools. Comparable clustering of both rearing conditions indicates consistent gene expression trajectories. PERMANOVA analyses confirmed significant stage differences across all gene sets ($p < 0.001$) with *post hoc* tests significant for all pairwise comparison ($p < 0.05$) except for vision/phototransduction genes between D1 and D3 ($p = 0.08$). (**B**) Temporal expression of genes encoding digestive. Y-axis shows $\log_2$ normalized counts + 1. Lines connect stage averages, and individual points show each sampled fish. Bolded genes are differentially expressed across developmental stages, revealing coordinated regulation of digestive capacity during post-settlement development.

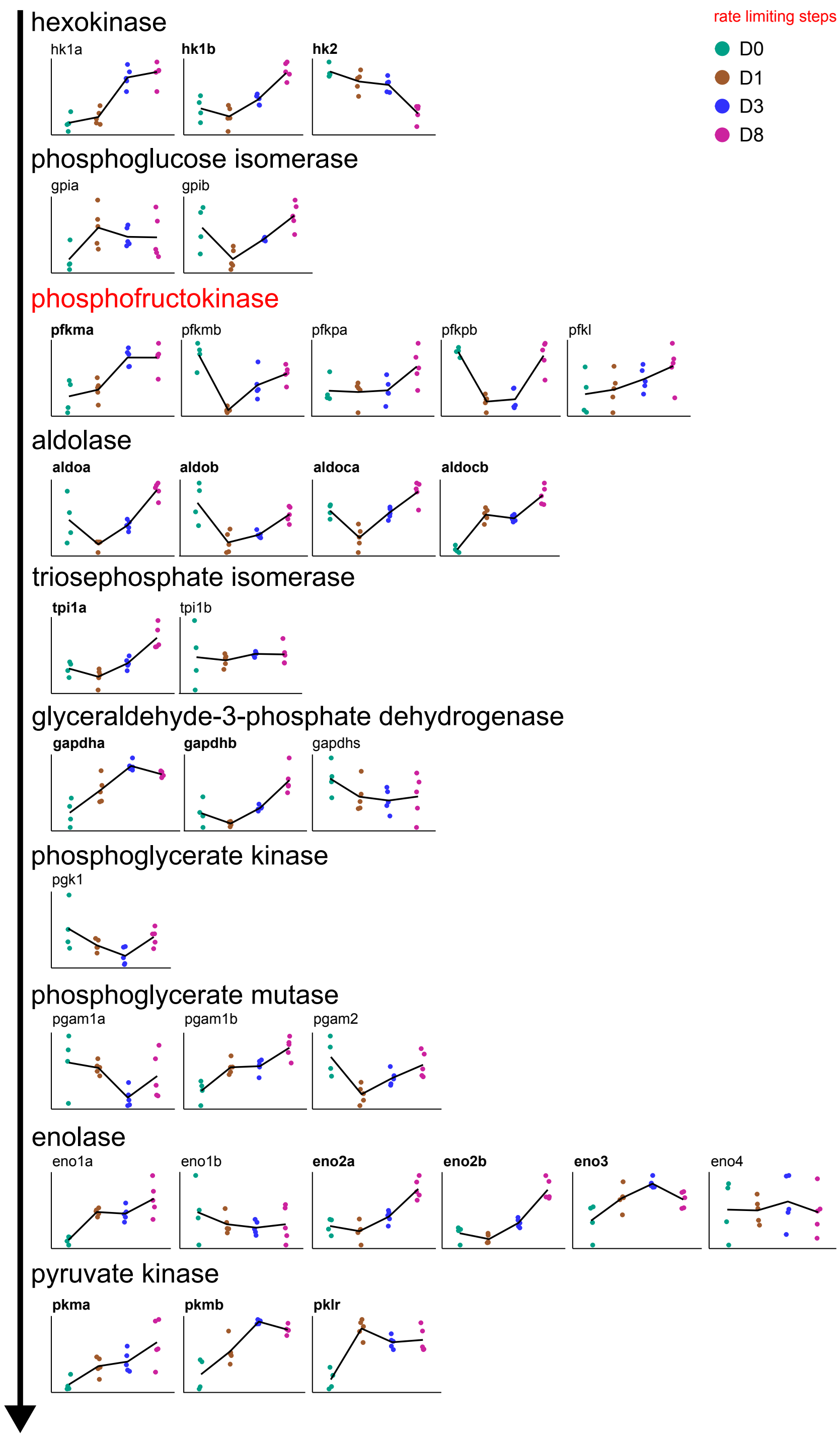

Fig. S2. Developmental modulation of glycolysis during post-settlement growth. Expression profiles of glycolytic pathway genes across four developmental stages in *Acanthurus triostegus*. The y-axis shows $\log_2$ normalized counts + 1, and the x-axis indicates developmental stage. Black lines connect averages; points represent individual fish. Genes in bold are differentially expressed over time, and genes in red denote rate-limiting steps, highlighting stage-specific modulation of glycolytic flux during early development.

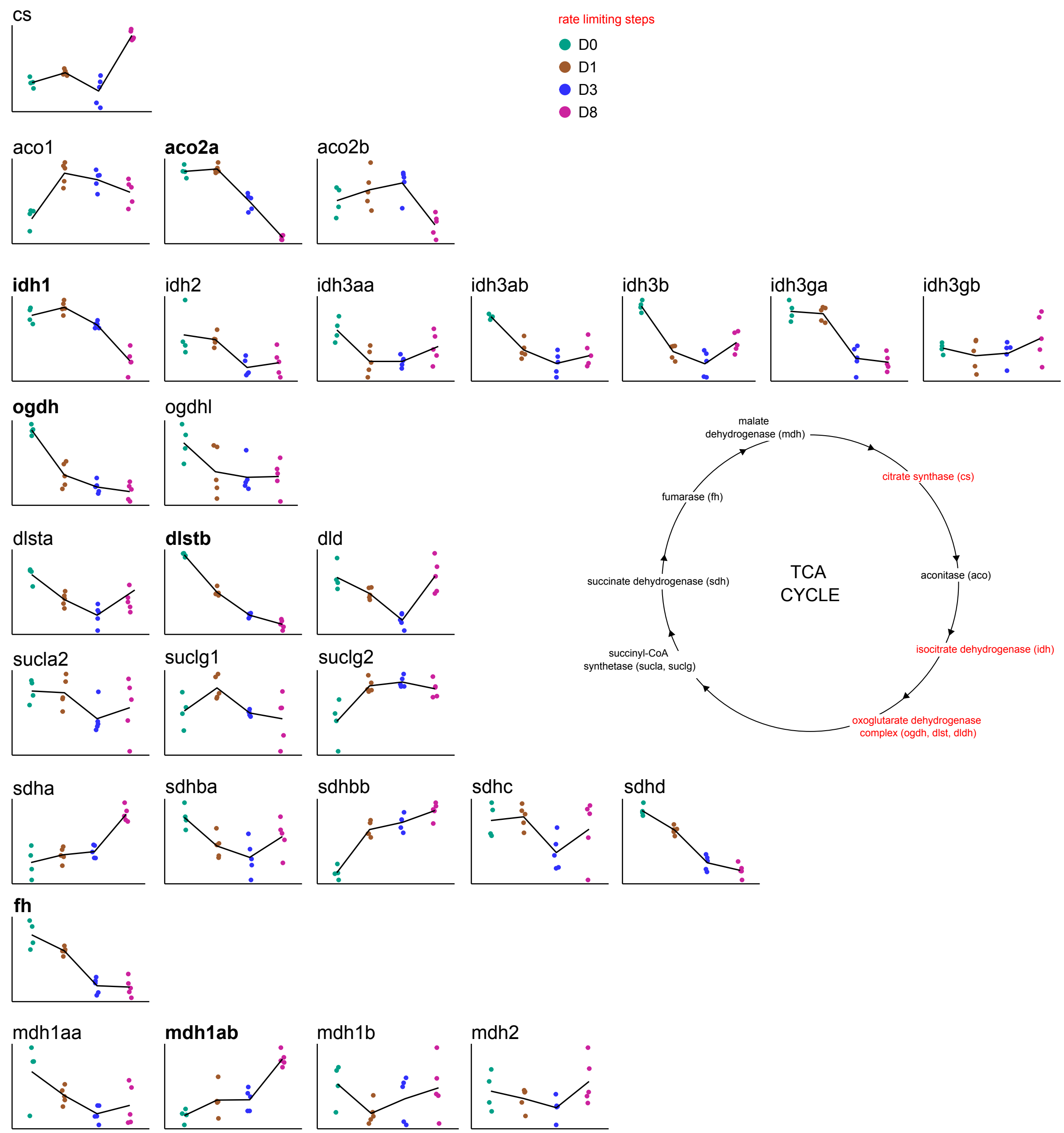

Fig. S3. Developmental regulation of the TCA cycle during post-settlement development. Expression profiles of tricarboxylic acid (TCA) cycle genes across four developmental stages in *Acanthurus triostegus*. Y-axis shows $\log_2$ normalized counts + 1, and the x-axis indicates developmental stage. Black lines connect stage averages; points represent individual fish. Genes in bold are differentially expressed over time, while genes in red denote rate-limiting steps, revealing stage-specific tuning of mitochondrial energy metabolism during early development.

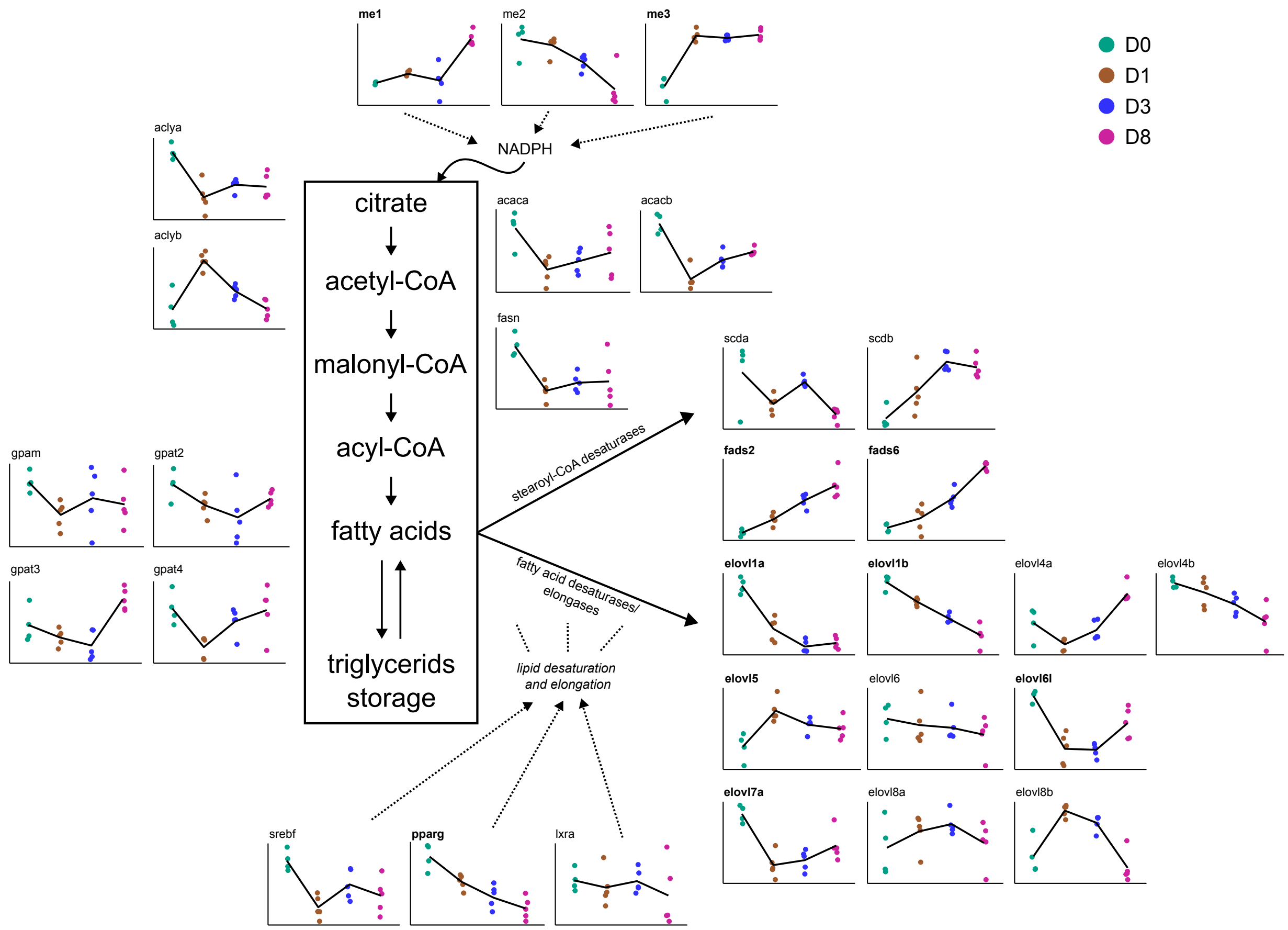

Fig. S4. Shifts in *de novo* lipogenesis during post-settlement development. Expression profiles of lipogenesis-related genes across four developmental stages in *Acanthurus triostegus*. Y-axis shows $\log_2$ normalized counts + 1, and the x-axis indicates developmental stage. Black lines connect stage average; points represent individual fish. Genes in bold are differentially expressed over time, and genes in red indicate rate-limiting steps, highlighting developmental regulation of lipid synthesis pathways. Adapted from (*24*).

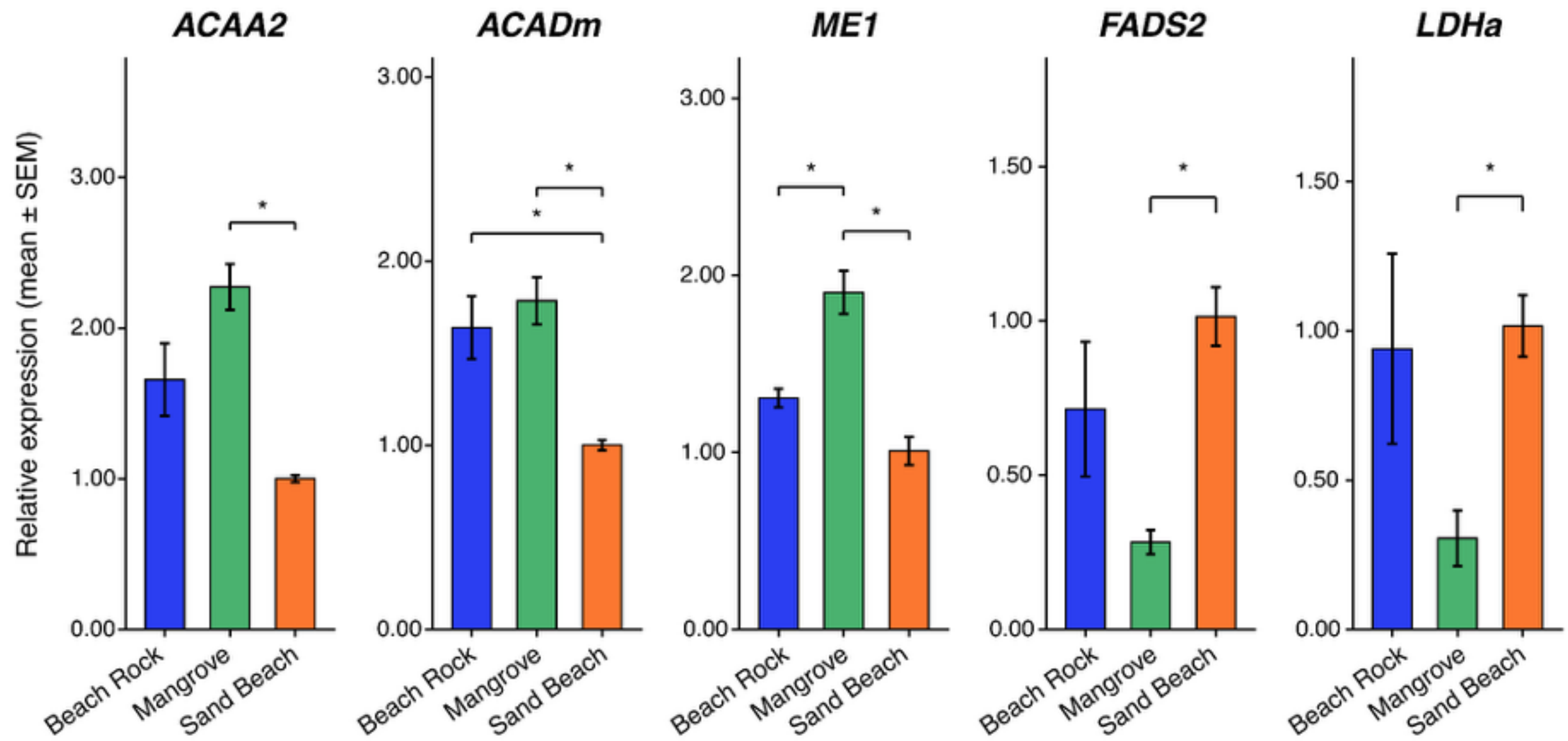

Fig. S5. Habitat-specific expression of key metabolic genes associated by qPCR. Relative expression of *ACAA2*, *ACADm*, *ME1*, *FADS2*, and *LDHa* in juveniles from beach rock (blue), mangrove (green), and sand beach (orange) habitats. Expression was normalized to *RPL7* and *POLD2* reference genes and calculated using the Pfaffl method with the sand beach group as reference. Error bars represent SEM. Asterisks indicate significant differences between groups (one-way ANOVA followed by Tukey HSD post-hoc test).

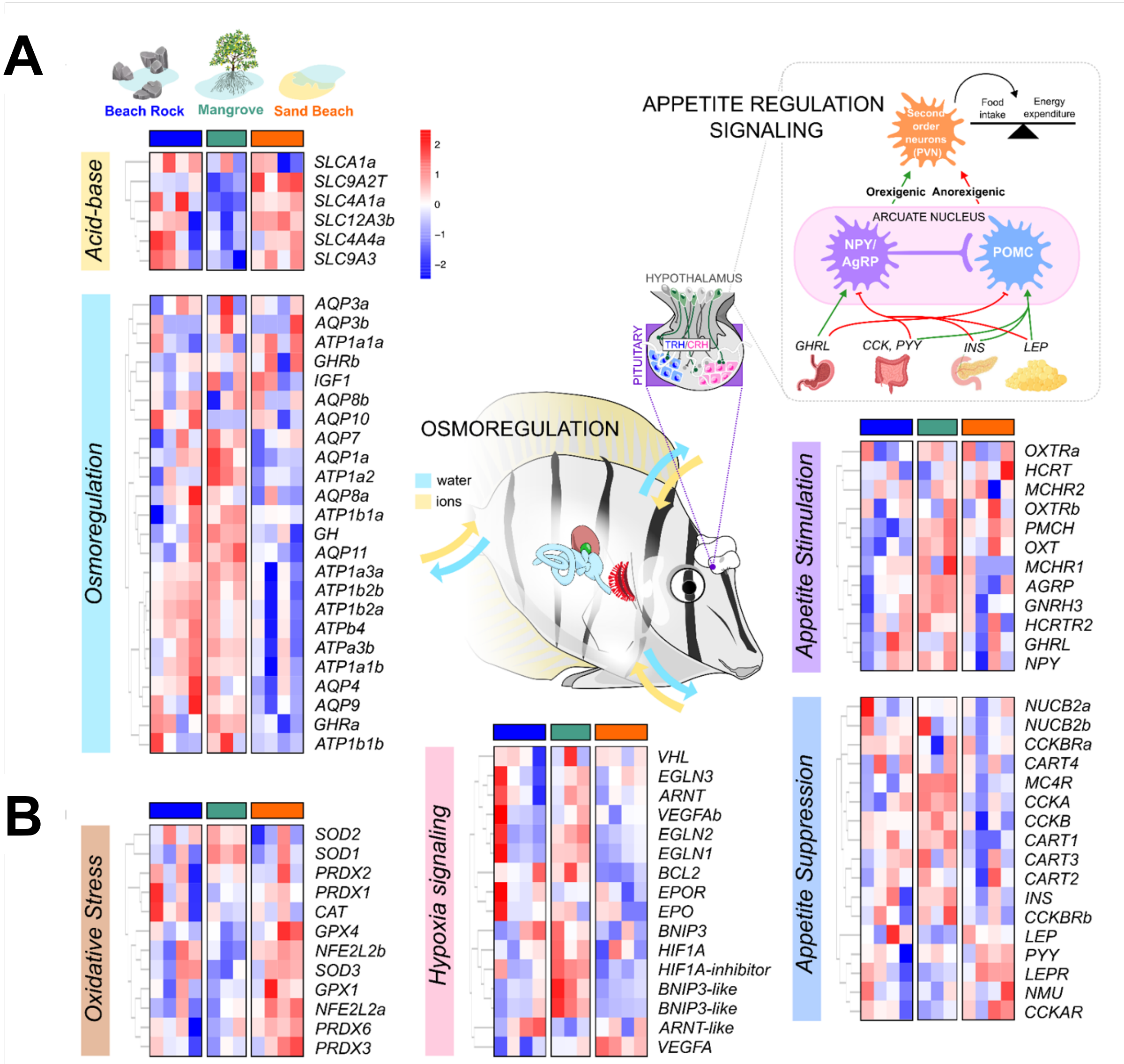

Fig. S6. Multidimensional hormonal plasticity supports local acclimation in *Acanthurus triostegus*. (**A**) *TRH* signaling regulates pituitary *TSH* cells and thyroid hormone production, while also promoting *GH* and *IGF-1* expression. Together with osmoregulatory proteins (aquaporins, acid-base transporters), these hormones maintain salinity and pH homeostasis under varying environmental conditions. A parallel *CRH*-mediated axis integrates peripheral signals (*LEP*, *GHRL*, *INS*) via arcuate nucleus neurons (*AGRP/NPY* and *POMC*), modulating appetite and energy balance through downstream neuropeptides (*AGRP*, *NPY*, *α-MSH*). (**B**) Endocrine regulation also supports responses to hypoxia and oxidative stress via *HIF* signaling and antioxidant enzymes. Heatmaps show z-scores of gene expression in wild-settled juveniles, with red indicating highly expressed genes and blue indicating low expression across habitats.

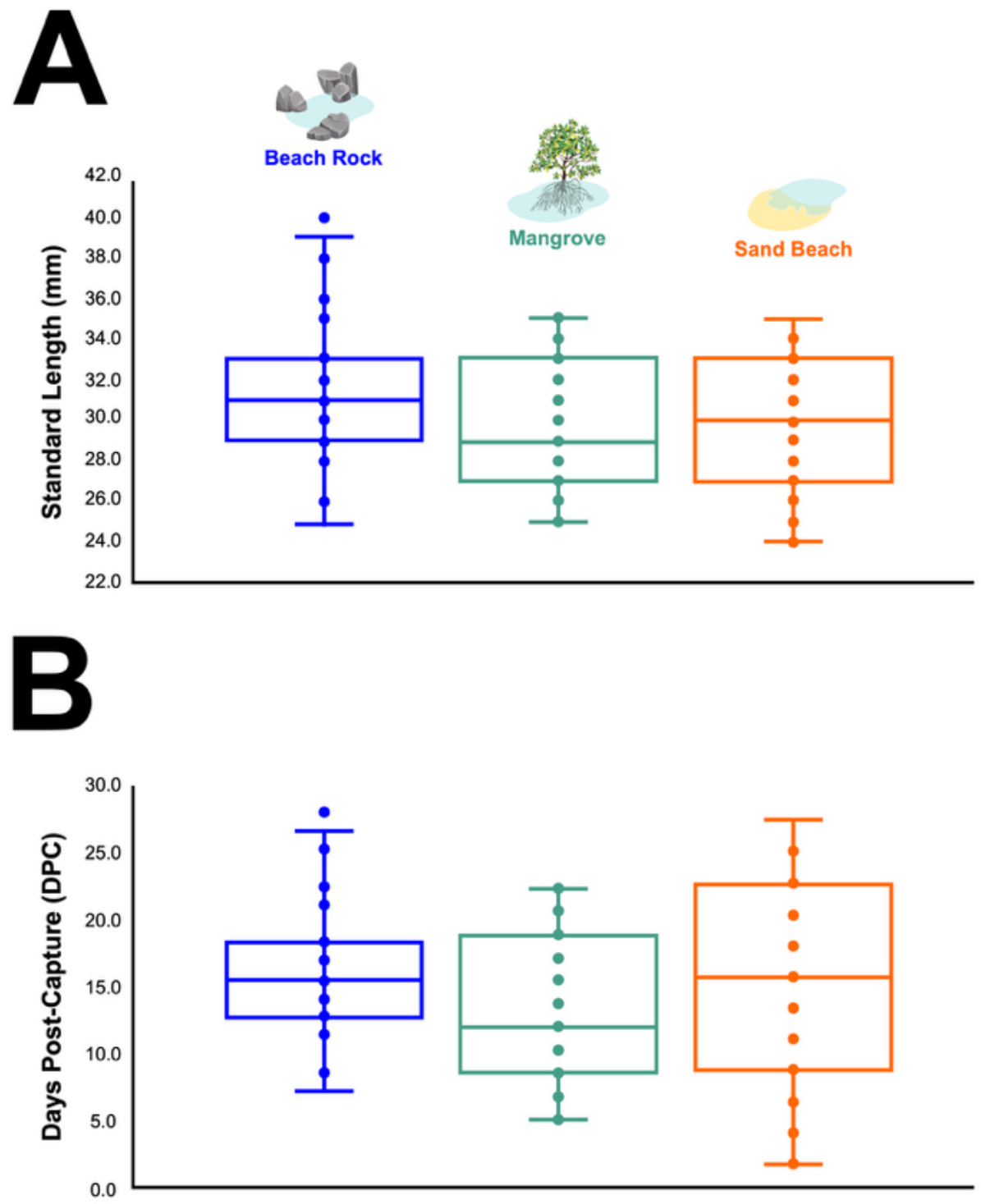

Fig. S7. Size and age of juvenile *Acanthurus triostegus* across nursery habitats. (**A**) Boxplots of standard length (mm) for juveniles from beach rock, mangrove, and sand beach habitats. (**B**) Estimated age (days post-capture) using habitat-specific growth regressions (Reynaud et al. (*20*)). Comparable distributions across habitats demonstrate that juveniles were size- and age-matched for all experiments (thyroid hormone measurements, RNA-seq, targeted metabolite profiling, and untargeted metabolomics). Boxplots show median, interquartile range, and 1.5× IQR whiskers.

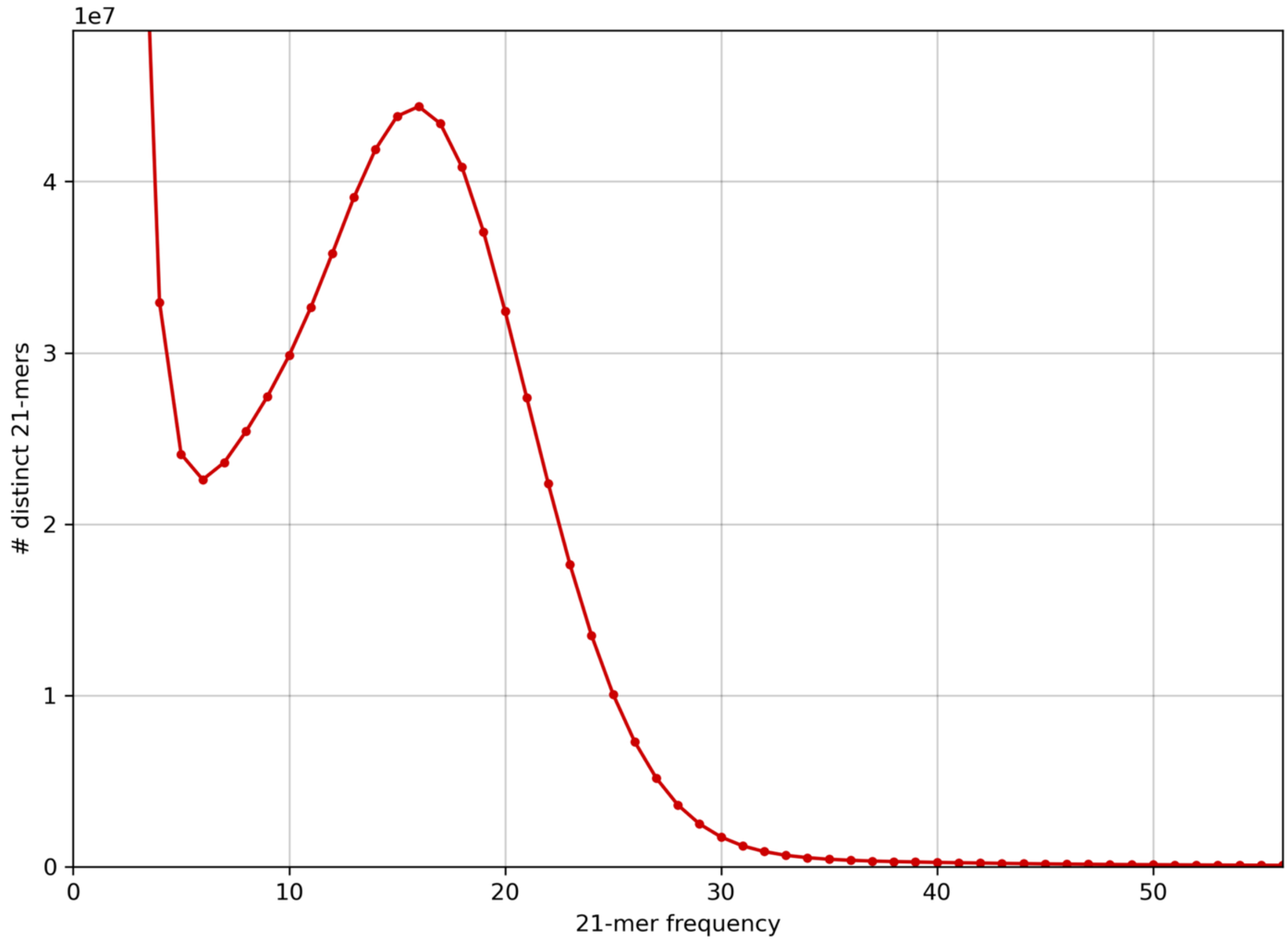

Fig. S8. 21-mer frequency spectrum of the *Acanthurus triostegus* genome. K-mer frequency distribution generated from ONT sequencing reads. The dominant peak at ~14× coverage represents homozygous genomic regions, while the lower-coverage shoulder (~7×) corresponds to heterozygous sequences. Low-frequency k-mers reflect sequencing errors, and the high-coverage tail indicates repetitive elements. The homozygous peak yielded a genome size estimate of 640.3 Mb and heterozygosity of 0.48%.

A

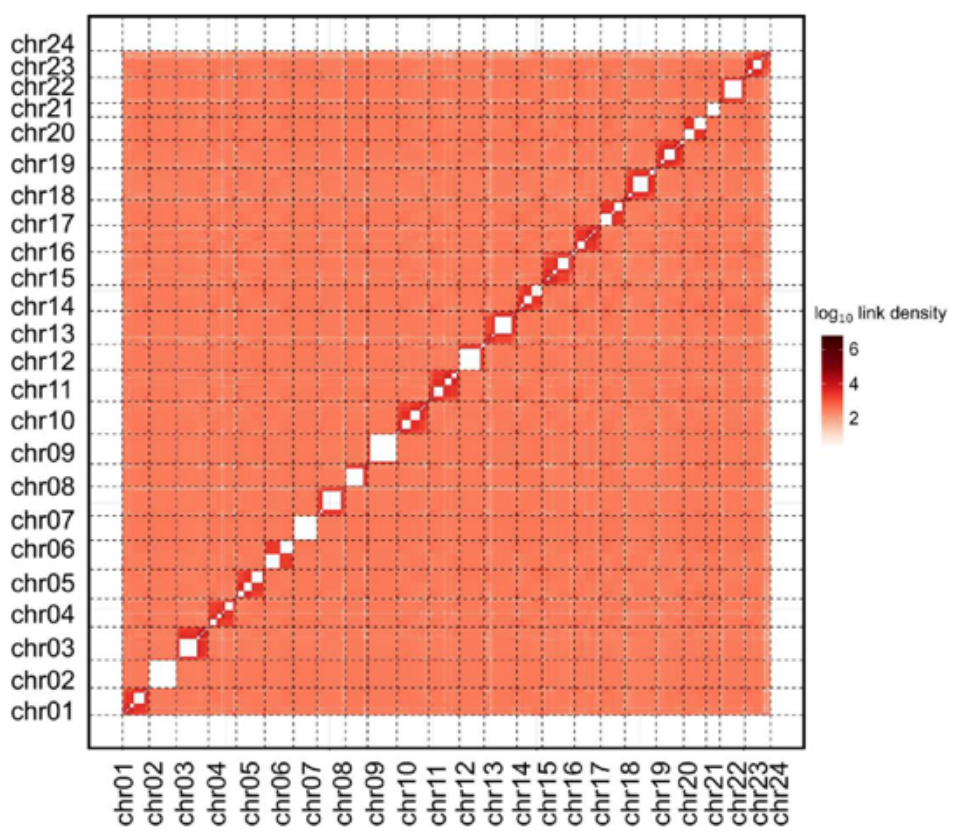

B

	Acanthurus triostegus (convict surgeonfish)	*Amphiprion ocellaris* (false clownfish)	*Chrysiptera cyanea* (blue damselfish)	*Epinephelus malabaricus* (Malabar grouper)
Sequency strategy	ONT MinION, Hi-C	PacBio Sequel II, Hi-C	PacBio Sequel II	PacBio Sequel II, Hi-C
Mean base-level coverage	29x	104x	94.5x	43x
Assembly size (Mb)	640.1	856.6	896.6	
Non-ATGC characters (bp)	40,100	136,641	not reported	36,700
GC content (%)	40.3	39.6	38.7	41.3
Repeat content (%)	38.0	44.7	36.0	56.4
Chromosome-scale N50 (Mb)	27.5	26.7	NA	Not reported
Contig N50 (Mb)	11.1	0.9	30.6	7.4
BUSCO genome completeness (%)	93.6	97.0	97.6	93.6
Complete and single copy (%)	92.7	96.0	96.9	92.3
Complete and duplicated (%)	0.9	0.8	0.7	1.3
Fragmented (%)	2.2	0.5	0.6	1.3
Missing (%)	4.2	2.5	1.8	5.1
No. protein coding genes	26,310	26,797	28,173	26,140
BUSCO gene annotation completeness (%)	94.7	96.6	96.6	95.5
Complete and single copy (%)	93.5	95.5	96.0	94.2
Complete and duplicated (%)	1.2	1.1	0.6	1.3
Fragmented (%)	2.5	1.0	1.5	0.9
Missing (%)	2.8	2.3	1.9	3.6
Data release	This study	Ryu *et al.* 2022	Gairin *et al.* 2024	Huerlimann *et al.* 2024

Fig. S9. Chromosome-level genome assembly of *Acanthurus triostegus*. (**A**) Hi-C contact map of the scaffolded genome assembly. Contact density ranges from dark red (high) to white (low), with both axes representing the 24 chromosomes. (**B**) Genome assembly statistics, gene annotation statistics, and BUSCO completeness for *A. triostegus* and other coral reef fish species.

Table S1. Gene Ontology (GO) terms enriched in each cluster corresponding to the four distinctive developmental stages sampled for *A. triostegus*. The numbers in brackets correspond to the GO terms displayed in the circular plots shown in Figure 1E. For each cluster, the number shown in the circular plot, the associated GO ID, the full GO term description, and the corresponding p-value are provided.

Table S2. Merqury-based assessment of base-level accuracy and k-mer completeness of the genome assembly. Base-level consensus quality and sequence completeness were evaluated using Merqury v1.3 (k = 21) based on Illumina-derived k-mers. Metrics are reported for the chromosome-scale assembly (all 24 chromosomes combined) and for unassembled contigs. Quality value (QV) represents consensus base accuracy on a Phred scale, the estimated error rate corresponds to the inferred per-base consensus error probability, and k-mer completeness indicates the proportion of reliable sequencing k-mers recovered in the assembly.

Table S3. Summary of RNA-seq sequencing quality control and alignment statistics for all samples. The table reports sample metadata (sample identifier, experiment, developmental stage, and habitat) together with sequencing and mapping metrics. Raw reads indicate the total number of sequencing reads generated per sample, and reads after trimming represent reads retained following adapter and quality filtering using Trimmomatic v0.39, with the percentage retained relative to raw reads. Alignment statistics were obtained from STAR v2.7.9a after mapping trimmed reads to the reference genome. Total aligned reads correspond to all reads successfully mapped to the genome, including both uniquely and multi-mapped reads. Uniquely mapped reads represent reads aligning of input reads mapped unambiguously. The multi-mapped reads (%) reflect reads aligning to multiple genomic loci (including reads mapped to multiple or too many loci), while unmapped reads (%) represent reads failing to align due to insufficient similarity, excessive mismatches, or insufficient aligned length. Percentages are calculated relative to the total number of reads after trimming.

Table S4. Sequences of primers used for qPCR validation of transcriptomic results, including reference genes used for normalization.
